# Reporting Quality of Social and Psychological Intervention Trials: A Systematic Review of Reporting Guidelines and Trial Publications

**DOI:** 10.1371/journal.pone.0065442

**Published:** 2013-05-29

**Authors:** Sean P. Grant, Evan Mayo-Wilson, G. J. Melendez-Torres, Paul Montgomery

**Affiliations:** 1 Centre for Evidence-Based Intervention, University of Oxford, Oxford, United Kingdom; 2 Centre for Outcomes Research and Effectiveness, Research Department of Clinical, Educational & Health Psychology, University College London, London, United Kingdom; University of Oxford, United Kingdom

## Abstract

**Background:**

Previous reviews show that reporting guidelines have improved the quality of trial reports in medicine, yet existing guidelines may not be fully suited for social and psychological intervention trials.

**Objective/Design:**

We conducted a two-part study that reviewed (1) reporting guidelines for and (2) the reporting quality of social and psychological intervention trials.

**Data Sources:**

(1) To identify reporting guidelines, we systematically searched multiple electronic databases and reporting guideline registries. (2) To identify trials, we hand-searched 40 journals with the 10 highest impact factors in clinical psychology, criminology, education, and social work.

**Eligibility:**

(1) Reporting guidelines consisted of articles introducing a checklist of reporting standards relevant to social and psychological intervention trials. (2) Trials reported randomised experiments of complex interventions with psychological, social, or health outcomes.

**Results:**

(1) We identified 19 reporting guidelines that yielded 147 reporting standards relevant to social and psychological interventions. Social and behavioural science guidelines included 89 standards not found in CONSORT guidelines. However, CONSORT guidelines used more recommended techniques for development and dissemination compared to other guidelines. (2) Our review of trials (n = 239) revealed that many standards were poorly reported, such as identification as a randomised trial in titles (20% reported the information) and abstracts (55%); information about blinding (15%), sequence generation (23%), and allocation concealment (17%); and details about actual delivery of experimental (43%) and control interventions (34%), participant uptake (25%), and service environment (28%). Only 11 of 40 journals referenced reporting guidelines in “Instructions to Authors.”

**Conclusion:**

Existing reporting guidelines have important limitations in content, development, and/or dissemination. Important details are routinely missing from trial publications; most leading journals in social and behavioural sciences do not ask authors to follow reporting standards. Findings demonstrate a need to develop a CONSORT extension with updated standards for social and psychological intervention trials.

## Introduction

Research in disciplines such as public health, psychology, education, social work, and criminology often involves complex interventions to improve health and related outcomes. Randomised controlled trials are increasingly used to evaluate these interventions and to inform decision-making in evidence-based policy and practice. However, these complex interventions have several unique features, such as multiple, interacting components (see Box 1)[1] that complicate critical appraisal of trial quality (e.g. risk of bias). Moreover, these interventions are often delivered in environments that are difficult to control and to measure, which makes reporting and interpretation of external validity (i.e., generalisability) difficult.[2]

High quality reports of complex intervention trials are important to diverse groups of stakeholders, including researchers, journal editors, funding agencies, practitioners, policy-makers, and research participants. These research consumers depend on accurate, complete, and transparent reports to appraise the validity and generalisability of trials. To address these needs, researchers and journal editors have developed reporting guidelines[3] that highlight key information about internal validity, external validity, and knowledge transfer of trials (e.g., locating trials in databases, assessing conflicts of interest). Reporting guidelines should consist of reporting standards (i.e., recommendations about the content that authors should consistently and transparently report) that are based on previous research and developed via expert consensus using rigorous, systematic, and transparent methodology.[4], [5]

The Consolidated Standards of Reporting Trials (CONSORT) Statement and its extensions are the preeminent guidelines for reporting trials. CONSORT is based on empirical evidence and expert consensus about biases related to trial validity.[6] Since its launch in 1996, CONSORT has had a considerable impact in the biomedical sciences; numerous reviews in the biomedical literature have shown an association between improvements in reporting quality and these guidelines.[7], [8]

Despite improvements in the completeness of RCT reports, major deficiencies in reporting quality still exist,[8] indicating that further actions are needed. For example, while CONSORT guidelines are well-known in the social and behavioural sciences, there is less evidence of widespread uptake and implementation in these disciplines compared with biomedical disciplines. Several studies also indicate that deficiencies persist in the reporting of social and psychological intervention trials.[9], [10], [11], [12], [13] A common explanation is that current standards in prominent reporting guidelines are not adequately tailored to these trials. For example, the CONSORT Statement and its extensions have primarily focused on standards related to internal validity, but researchers are increasingly interested in the applicability of trial findings and have called for updated standards to improve the assessment of external validity.[14], [15], [16], [17], [18], [19] For example, researchers have asked for more information related to process evaluations, such as intervention theory of change, assessment of intervention mechanisms during the trial, and relevant information about the influence of trial context.[14]–[16] To determine whether a new reporting guideline is needed, it is necessary (i) to assess the suitability of current reporting guidelines for social and psychological intervention trials and (ii) to investigate the quality of reports of these trials.

## Objectives

Following recommended techniques for guideline development and dissemination,[3] a structured approach to reporting guideline development should begin with a needs assessment that (i) reviews whether an adequate guideline already exists for a given research method and (ii) obtains evidence of the reporting quality of published research using that method.[4] Though highly informative, previous reviews have not investigated the characteristics and methods of development of reporting guidelines specifically for social and psychological intervention trials. Moreover, previous reviews about the reporting quality of these trials have consisted of small samples and have assessed reporting quality according to a narrow set of reporting standards.[12], [13], [20]

We conducted a two-part study that examined:

1. the content, development, and dissemination of current reporting guidelines; and
2. the current reporting quality of social and psychological intervention trials across several disciplines according to a comprehensive set of reporting standards.

## Methods

### Eligibility Criteria

For the first part of the study, a reporting guideline had to consist of a published, peer-reviewed article that introduced a formal, itemised checklist of reporting standards relevant to trials of social and psychological interventions. In order to identify all published and potentially relevant reporting standards, quality assessment tools (e.g., tools designed to be used for critical appraisal) were also eligible. For practical reasons, we limited the search to guidelines available in English.[5] We excluded guidelines for the *design and conduct* of trials rather the *reporting* of trials, as well as tools pertaining to a specific intervention focus that is unrelated to social and psychological interventions (e.g., acupuncture, complementary medicine).

For the second part of the study (i.e., the review of trial reporting quality), a trial report had to discuss a randomised experiment of a complex intervention with psychological, social, or health outcomes. We excluded trial reports that: (i) described process evaluations without trial outcomes, (ii) evaluated only cost-effectiveness, (iii) used randomisation to balance order of exposure to conditions that were experienced by all participants, or (iv) explicitly evaluated medical or pharmacological interventions. No other eligibility criteria were used.

### Search Strategy and Study Selection

For the first part of the study, we used an adapted version of a peer-reviewed electronic search strategy[21] to identify relevant reporting guidelines (see Text S1). We also searched three registries of reporting guidelines: the EQUATOR Network library of identified health research reporting guidelines (www.equator-network.org), a recent review on the development and contents of reporting guidelines for health research,[21] and a systematic review of studies assessing the quality of conducting or reporting trials.[22] We also searched references of all eligible guidelines identified through this process.

For the second part of the study, we conducted a hand search of journals' Table of Contents throughout the year 2010. From the ISI Web of Knowledge 2010 Journal Citation Reports (JCR) for Social Sciences, we identified journals publishing trials of complex interventions in clinical psychology, criminology, education, and social work. To obtain an extensive sample of trials, we searched the 10 journals with highest impact factors in each field (40 journals total) that published trials in the year 2010.

### Data Abstraction

We first examined the content of reporting guidelines by compiling reporting standards from all identified guidelines into a comprehensive, non-redundant, itemised list of standards (see Appendix S1).[6] To assess the quality of reporting guideline development, we compared the techniques used by guideline developers to recommended techniques,[3], [21] which were organised according to four phases of process: preliminary work, development of the guideline itself, publication, and dissemination activities (see Appendix S2). One reviewer (SG) assessed whether guidelines adhered to each standard.

We assessed guideline dissemination in several ways. Akin to previous studies,[4] we performed a full-text review of each journal's “Instructions to Authors” to identify references to guidelines for reporting trials (e.g., instructions on the journal webpage, mention of a reporting guideline) and whether journals required authors to register trial protocols before recruiting participants. For each guideline, we also counted citations through November 2012 using Google Scholar, which provides a wide measure of impact across most publication mediums.[23] If a guideline was published in multiple journals or included an official explanatory document detailing how to adhere to its reporting standards, we combined the citations for all documents.

To assess the reporting quality of identified trials, two reviewers (SG and GJMT) independently assessed whether trial reports adhered to each standard in our comprehensive list of relevant reporting standards (Appendix S1). As the goal was to identify potential limitations in both guidelines and reporting quality, we used a comprehensive checklist to assess trial reports according to all published and potentially relevant reporting standards rather than a single instrument (such as the CONSORT Statement). Coding rules were adapted from previous studies about trial reporting quality.[9], [24]

Before assessing the entire sample, the reviewers coded one trial report in each discipline and compared results to ensure consistent application of coding rules. The two reviewers then each coded the entire sample. Discrepancies in judgment were resolved through discussion and consensus. Using SPSS version 18, inter-rater agreement prior to discussion and consensus was calculated as κ = 0.71, indicating substantial agreement.[25] Data resolved after discussion were used for the final analyses.

### Data Analysis

Similar to previous studies,[9], [24] we analysed guideline content by mapping identified reporting standards onto standards included in the CONSORT Statement in order to organise the checklist according to the common sections of a trial report (i.e. introduction, methods, results, and discussion). We also noted any reporting standards that are not in official CONSORT guidelines but were found in other guidelines. We summarised adherence to recommended techniques for reporting guideline development as frequencies,[3], [21] and we converted total citations of each guideline into median citations per year. Data about the development and dissemination of guidelines were compared by the following pre-specified types of reporting guideline: official CONSORT guidelines, non-CONSORT guidelines for medical sciences, or non-CONSORT guidelines for social and behavioural sciences.

To describe the quality of trial reports, we summarised adherence to reporting standards as frequencies.[21] We analysed compliance to reporting standards for the whole sample and by academic discipline to provide a preliminary view of differences in reporting across social and behavioural sciences. We also categorised reporting standards into *a priori* conceptual themes often targeted by reporting guidelines: internal validity, external validity, and other important study details (e.g., information for indexing and certain ethical concerns).

## Results

### Previous Guidance

**Figure 1:**
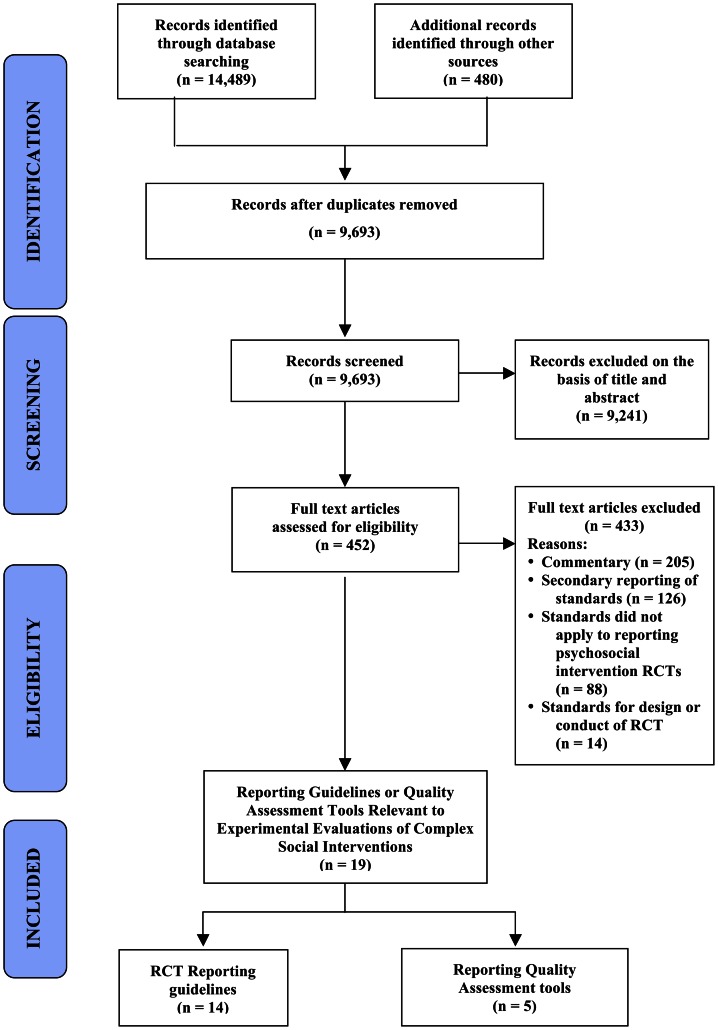
Flowchart of reporting guidelines through systematic literature search.

Through the literature search (see Figure 1), we identified 19 unique, eligible reporting guidelines and reporting quality assessment tools (see Table 1) developed between 1980 and 2010 (median 2004).[6], [9], [26], [27], [28], [29], [30], [31], [32], [33], [34], [35], [36], [37], [38], [39], [40], [41], [42] Six were developed by the CONSORT Group for reporting RCTs; six were non-CONSORT documents for health-research trials in general; and seven were specific to research in the social and behavioural sciences, namely non-randmoised trials of public health interventions,[38] empirical research in education,[27] empirical research in psychology,[41] trials in criminal justice,[9] outcome studies of alcohol treatment,[26] trials in occupational therapy,[24] and the content of behavioural change interventions.[39]

**Table 1:** Characteristics of included reporting guidelines and reporting quality assessment tools.

Guideline	Year	Document Type	Official CONSORT	Targeted Area	# Reporting Standards
Reporting Guidelines Specific to the Social and Behavioural Sciences					
Alcohol Outcome Studies Coding Sheet [26]	2010	AT		Alcohol	36
AERA Standards for Empirical Social Science Research [27]	2006	RG		Education	56
CONSORT and Criminal Justice Trials (CJT) Project Coding Sheet [9]	2010	AT		Criminology	54
Journal Article Reporting Standards [41]	2008	RG		Psychology	134
Nelson-Moberg Expanded CONSORT Instrument [34]	2004	AT		Occupational Therapy	201
TREND Statement [38]	2004	RG		Public Health	59
WIDER [39]	2009	RG		Behavioural Change Interventions	12
Other Reporting Guidelines					
CONSORT Extension for Abstracts [28]	2008	RG	x	Abstracts	17
CONSORT Extension for Cluster Trials [29]	2004	RG	x	Cluster Trials	40
CONSORT Extension for Non-Pharmacological Treatments [30]	2008	RG	x	Non-Pharmacological Interventions	27
CONSORT Extension for Pragmatic Trials [31]	2008	RG	x	Pragmatic Trials	25
CONSORT Extension for Reporting Harms [32]	2004	RG	x	Harms	22
CONSORT Statement [6]	1996	RG	x	None	37
Evidence-Based Behavioral Medicine-Specific Guidelines [33]	2003	RG		Behavioural Medicine	34
Jadad Scale [40]	1996	AT		None	3
Oxford Implementation Index [35]	2007	AT		Complex Interventions	17
Quality Evaluation Form [36]	1995	AT		None	20
Reporting Standards for Controlled Trials [42]	1980	RG		None	6
Structured Reporting of Randomized Controlled Trials [37]	1994	RG		None	32

In “Document Type” column, AT = reporting quality assessment tool, and RG = reporting guideline. In “Official CONSORT” column, a “x” means that the guideline is an official CONSORT guideline.

Overall, CONSORT guidelines used recommended techniques for guideline development and dissemination more frequently than non-CONSORT guidelines in medical, social, and behavioural sciences (see Table 2). Notably, most CONSORT guidelines tended to use more rigorous consensus methods in the development stage (75% of recommended techniques) compared with medical guidelines (44%) and social and behavioural science guidelines (37%), such as formal consensus development processes (see Table S1). Most CONSORT guidelines adhered to most dissemination activities (77%), such as endorsement and adherence by journals, while most other medical guidelines (10%) and social and behavioural science guidelines (34%) did not. In addition, CONSORT guidelines were cited more often (74 citations per year) than other guidelines in medicine (10) or social and behavioural sciences (4).

**Table 2:** Average percentage of recommended techniques for guideline development by document type.

Guideline Development Stage	CONSORT (n = 6)	Non-CONSORT Medical (n = 6)	Social & Behavioural Science (n = 7)
			
1. Preliminary Activities	91.7%	70.8%	67.9%
2. Document Development	75.0%	44.4%	31.0%
3. Publication Strategy	66.7%	5.5%	23.8%
4. Dissemination	76.7%	10.0%	37.1%
Median Citations per Year (*Range*)	73.7 (*43.3 – 535.5*)	9.9 (*0.2 – 480.2*)	4.4 (*1.0 – 65.0*)

Citation count derived from Google Scholar search on 1 November 2012.

Stage 1 = 4 items, Stage 2 = 6 items, Stage 3 = 3 items, Stage 4 = 5 items

The 19 included reporting guidelines included a median of 32 reporting standards (interquartile range (IQR) = 17 to 54; range = 3 to 201) From these, we developed a list of 147 non-redundant reporting standards that are relevant to social and psychological interventions (see online Appendix S1). Of these 147 reporting standards, 89 were either not included in CONSORT guidelines or were tailored versions of CONSORT standards for social and psychological interventions (see Table S2 for a full list). Amongst these standards, requests for details about setting, implementation of the interventions, data collection, generalisability, and ethical concerns were common.

### Assessment of Reporting Quality

Only 11 of the 40 journals referenced a published reporting guideline in their “Instructions to Authors” section (see Table 3). Two journals provided advisory text about reporting certain aspects of intervention studies without reference to any published reporting guideline; no other journals provided any textual instructions specific to reporting trials. Only 5 journals required trials to be registered in a trial registry (e.g., clinicaltrials.gov) prior to publication.

**Table 3:** Sample of journals included in reporting quality review.

Journal	ISI 2010 Impact Factor	Reporting Guidance Specific to RCTs in “Instructions to Authors”	Trial Registration Required	Eligible RCTs in 2010
Clinical Psychology				
*Archives of Sexual Behavior*	3.660	None	No	2
*Health Psychology*	3.982	CONSORT; JARS	Yes	16
*Journal of Abnormal Child Psychology*	3.564	None	No	7
*Journal of Abnormal Psychology*	5.235	JARS	No	1
*Journal of Behavioral Medicine*	3.232	CONSORT; TREND	No	14
*Journal of Clinical Child and Adolescent Psychology*	3.440	CONSORT; JARS	Yes	8
*Journal of Clinical Psychiatry*	5.023	Text about reporting intervention studies	Yes	5
*Journal of Consulting and Clinical Psychology*	5.227	JARS	No	35
*Neuropsychology*	3.176	CONSORT; JARS	Yes	2
*Psychological Medicine*	5.200	None	No	9
Criminology				
*British Journal of Criminology*	1.612	None	No	1
*Crime & Delinquency*	1.750	None	No	1
*Criminal Justice and Behavior*	1.590	None	No	4
*Criminology*	2.658	None	No	1
*International Journal of Offender Therapy and* *Comparative Criminology*	1.071	None	No	2
*Journal of Criminal Justice*	1.076	None	No	3
*Journal of Interpersonal Violence*	1.354	None	No	6
*Justice Quarterly*	1.211	None	No	1
*Psychology, Crime & Law*	1.133	None	No	11
*Youth Violence and Juvenile Justice*	1.132	None	No	1
Education				
*American Educational Research Journal*	2.479	AERA	No	3
*Computers & Education*	2.617	None	No	39
*Early Childhood Research Quarterly*	2.192	Text about reporting effect sizes	No	4
*Educational Evaluation and Policy Analysis*	1.919	AERA	No	1
*Journal of Engineering Education*	2.219	None	No	7
*Journal of Research in Science Teaching*	2.728	None	No	7
*Journal of Teacher Education*	1.891	None	No	3
*Learning and Instruction*	2.768	None	No	19
*Metacognition and Learning*	2.038	None	No	2
*Science Education*	1.900	None	No	4
Social Work				
*American Journal of Community Psychology*	1.722	JARS	No	1
*Child Abuse & Neglect*	1.945	None	No	2
*Child Maltreatment*	1.984	None	No	2
*Children and Youth Services Review*	1.130	None	No	3
*Family Relations*	1.216	None	No	2
*Health & Social Care in the Community*	1.008	CONSORT; TREND	Yes	1
*Health & Social Work*	1.143	None	No	1
*Journal of Community Psychology*	0.792	None	No	1
*Research on Social Work Practice*	1.130	JARS	No	6
*Social Service Review*	1.421	None	No	1

Reporting Guidance Specific to RCTs in “Instructions to Authors”: whether the “Instructions to Authors” section of a journal provided any guidance or referred to any guidelines on reporting RCTs. Trial Registration Required: whether the journal required RCTs to be registered in a trial registry (e.g., clinicaltrials.gov) prior to publication. Eligible RCTs in 2010: number of RCTs in 2010 that met eligibility criteria

**Figure 2:**
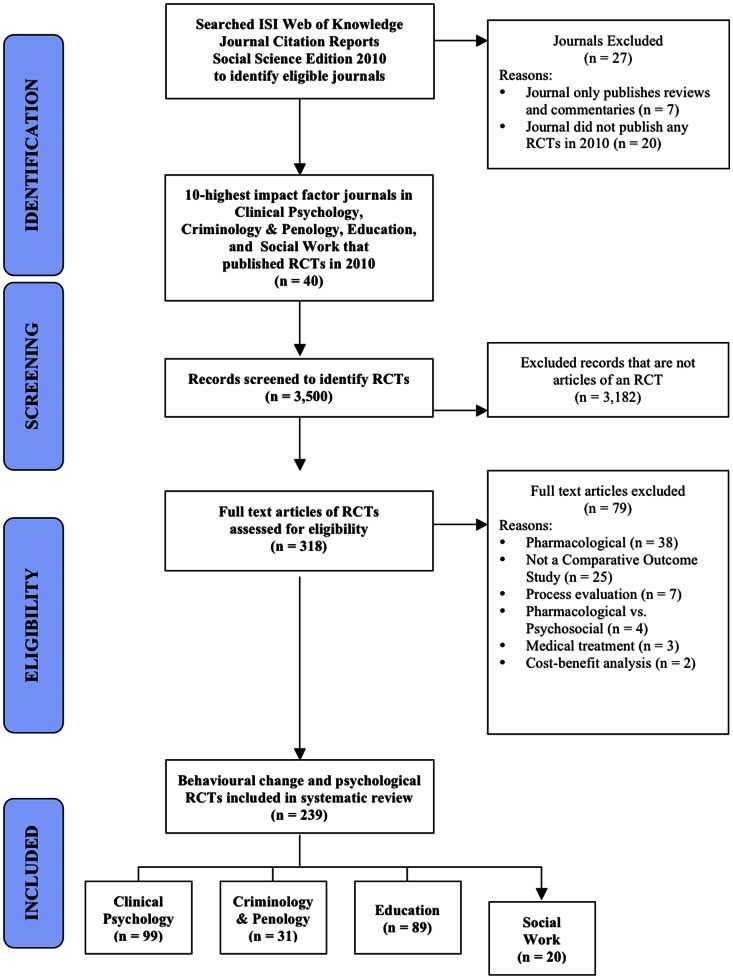
Flowchart of considered RCT publications through systematic literature search.

From these journals, we identified 239 eligible trials (Figure 2), including between 1 and 39 per journal (median 3). Overall, trials reported a mean of 42% of all reporting standards; there was low compliance with reporting standards related to internal validity (38%), external validity (47%), and other study details (34%). Reporting quality did not vary substantially by discipline (Table 4).

**Table 4:** Average compliance of RCTs with reporting standards.

Area	Item	Clinical Psychology	Criminology	Education	Social Work	Total Sample
External Validity						
10 Items	Participants	54.6%	38.2%	37.9%	53.2%	46.2%
7 Items	Timing and Setting	43.1%	46.5%	44.8%	55.7%	45.2%
29 Items	Intervention: Average	50.4%	42.8%	52.4%	48.3%	50.0%
10 Items	*Intervention Implementation: Design*	74.1%	69.7%	79.7%	80.0%	76.1%
12 Items	*Intervention Implementation: Delivery*	43.8%	35.5%	44.8%	37.9%	42.6%
7 Items	*Intervention Implementation: Uptake*	27.8%	17.1%	26.5%	20.7%	25.3%
26 Items	Control: Average	38.4%	38.0%	46.9%	22.1%	40.1%
8 Items	*Control Implementation: Design*	60.5%	62.1%	70.9%	43.1%	63.1%
12 Items	*Control Implementation: Delivery*	32.3%	31.5%	41.4%	16.2%	34.2%
6 Items	*Control Implementation: Uptake*	21.0%	18.8%	25.8%	5.8%	21.3%
2 Items	Programme Differences	29.8%	27.4%	27.0%	17.5%	27.4%
4 Items	Outcomes*	67.2%	54.8%	53.7%	56.3%	59.6%
5 Items	Interpretation	75.6%	58.7%	51.2%	63.0%	63.3%
83 Items	Total External Validity	48.4%	42.2%	47.7%	41.8%	46.8%
Internal Validity						
9 Items	Trial Design	58.7%	50.9%	50.3%	57.2%	54.4%
4 Items	Random Sequence*	30.1%	11.3%	18.0%	28.8%	23.0%
13 Items	Data Analysis*	50.0%	31.8%	36.0%	44.6%	41.9%
3 Items	Allocation Concealment*	26.3%	17.2%	3.4%	28.3%	16.7%
3 Items	Blinding*	20.2%	4.3%	11.2%	18.3%	14.6%
8 Items	Participant Flow*	55.4%	14.5%	20.4%	37.5%	35.6%
40 Items	Total Internal Validity	47.0%	27.4%	30.0%	41.2%	37.6%
Study Details						
16 Items	Title and Abstract	40.8%	17.9%	28.4%	34.7%	32.7%
5 Items	Protocols and Manuals*	29.9%	11.6%	14.6%	27.0%	21.6%
3 Items	Ethical Concerns	78.1%	47.3%	41.9%	76.7%	60.5%
24 Items	Total Study Details	43.2%	20.3%	27.2%	38.3%	33.9%
Total Score						
	Total Score for All Standards	47.2%	34.6%	39.5%	41.1%	42.2%

Number of RCTs in each discipline: RCTs per discipline: Clinical Psychology—99, Criminology—31, Education—89, Social Work—20

* Denotes Cochrane Risk of Bias item

**Figure 3:**
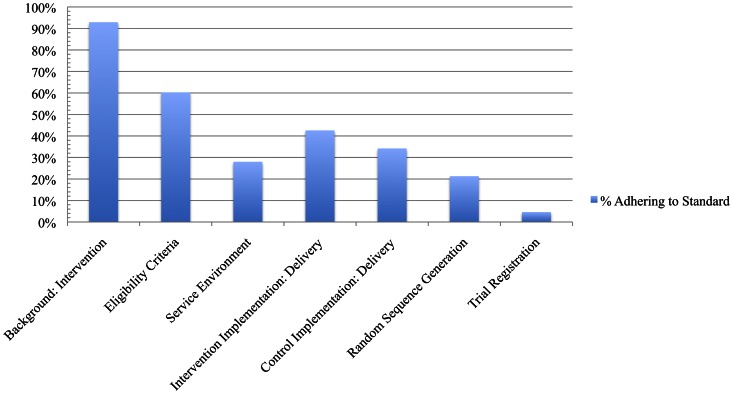
Average compliance of RCTs with key reporting standards.

Several important aspects of trials were not consistently reported and would be easy to include in all trial reports (see Table S3; Data File S1). Only 20% of reports identified the trial as randomised in the title, and only 55% identified the trial as randomised in the abstract. Overall, 60% of reports included the trial eligibility criteria, but the majority of these reports did not explicitly list all inclusion *and exclusion* criteria. Trial reports adhered to only 23%, 17%, and 15% of the standards related to random sequence generation, allocation concealment, and blinding respectively. While most reports (71%) included the number of participants randomised to each condition, few reports described other aspects of participant flow through the trial, such as the number of participants: eligible for the trial (33%), receiving treatment (31%), and included in the primary analyses (38%). Less than half of the reports reported primary outcomes (27%) or secondary outcomes (45%) sufficiently to be included in meta-analyses. Very few reports (5%) indicated that the trial had been registered, and few reports included information about a trial protocol (8%) or access to a treatment manual (40%). Reports adhered to 50% of standards related to the implementation of the intervention and included a mean of 28% of standards related to the context of the wider service environment (see Figure 3).

## Discussion

### Overall Findings

Results establish the need for a new reporting guideline. This review identified numerous guidelines that have made useful contributions to reporting medical and social research. However, this study demonstrates that current reporting guidelines are insufficient for social and psychological intervention trials. Compared with the CONSORT Statement and its official extensions, guidelines in the social and behavioural sciences have not consistently followed recommended techniques for development and dissemination,[3] and they have not been widely utilised. If not properly developed and disseminated, these guidelines are potentially of limited use and are less likely to improve reporting of key features of trials that are important to stakeholders.[5] However, these guidelines include important, tailored standards for social and psychological interventions that are not found in CONSORT guidelines. Due to the substantial variability of recommended standards across reporting guidelines, disseminating CONSORT or another guideline would insufficiently address social and psychological intervention trials; further work is required to improve the applicability, utility, and acceptability of reporting guidelines in disciplines outside medicine.

Our analysis of trial reporting quality suggests that trial reports often fail to comply with published reporting standards, including well-established standards in the CONSORT Statement and its extensions. While reporting quality varies across standards and disciplines, this review shows that most trial reports omit information that is necessary to assess internal and external validity. This finding is consistent with previous studies of reports of social and psychological intervention trials in specific disciplines.[9], [10], [11], [12], [13] Poor reporting also has serious implications for knowledge transfer. For example, reports that are not identified as randomised trials in their titles or abstracts may not be identified in electronic literature searches and may be omitted from reviews as a result. The development and dissemination of a tailored reporting guideline could help resolve these problems.

### Strengths and Limitations of the Current Study

This study is the most comprehensive review of reporting guidelines and the reporting quality of social and psychological intervention trials ever conducted. We undertook a highly sensitive search for reporting guidelines and assessed their use across numerous journals in several disciplines. We also conducted a complete assessment of all trial reports in 40 leading journals in one year, double coded their reporting quality, indicated clustering of reporting quality by journals within disciplines, and utilised a comprehensive set of standards to prevent selective assessment and reporting of quality.[8] While the reviewers weren't blind to the authors, institutions, and journals of RCT reports due to resource restraints, there is currently no evidence to suggest that such lack of blinding the validity of these reviews assessing reporting quality.[8]

It is clear that reporting guidelines for trials are not widely used outside medicine, but there may be several reasons for this. Regardless, social and behavioural scientists have been aware of the CONSORT Statement and its extensions for some time, so lack of uptake is not the result of ignorance of these guidelines.[14], [15], [43] Our correspondence with journal editors confirmed that many are familiar with CONSORT and some related guidelines.

In assessing compliance with reporting standards included in these guidelines, we found that several standards are vague and underdeveloped, particularly those related to external validity, such as theory of change.[14] When standards are imprecise, reports can be compliant without describing evaluations sufficiently to allow critical appraisal, replication, and inclusion in reviews and meta-analyses. Moreover, though inter-rater agreement in the review of reporting quality was high (κ = 0.71), it did not reach newly-developed criteria (κ≥0.80) for assessing the validity of evaluations of RCTs reporting quality.[8] Our own difficulty in applying some standards reaffirmed the need to develop clear, specific recommendations for social and psychological intervention trials based on best current evidence.[18]

Despite the difficulties in developing a comprehensive set of reporting standards, deficiencies in trial reports are both real and important. We included trial reports that are most likely to be cited (i.e., those published in high impact journals) and which may be of better quality than articles published in low impact journals.[4], [44] The reports assessed are probably representative of the best trial research in these disciplines.

### Future directions

A reporting guideline designed specifically for social and psychological interventions would help improve the quality of these trial reports.[43], [45] To be acceptable and widely utilised, such a guideline should be developed using rigorous methods that engage members from all relevant stakeholder groups during development and dissemination, and its reporting standards should be based on sound empirical evidence where possible.[3], [5] Given the prominence of CONSORT internationally, the precedence of its standards, and the rigorous development and dissemination practices of the CONSORT Group, an official CONSORT extension seems the best method to facilitate better reporting of these trials.

This study identified many new and modified reporting standards that could be added to the CONSORT Statement to form an official extension. Several standards in current CONSORT guidelines could be amended to make them more applicable and acceptable for trials of social and psychological interventions. For example, modifications could attend to difficulties in: blinding participants and providers of complex interventions, participant and provider preferences, the use of multiple measurement formats (e.g., self-report, observation) within a study, and the complexity of data analysis.[9], [14], [33], [34], [41], [46] In addition, researchers are increasingly demanding better reporting standards related to external validity, theory of change, and implementation.[1], [14], [19] Standards in guidelines other than the CONSORT Statement include relatively more information about sample characteristics,[35], [47] the extent to which trials differ from usual practice,[27] details about facilitative or obstructive aspects of the trial context,[48] and contextual factors related to feasibility and coverage,[14] such as organisational resources and the wider service system structure.[49], [50] Such information is important to improve the knowledge base for effective transfer of research findings to real-world settings.[51] Details of trials not related to internal and external validity are also important, such as discussing other relevant research when interpreting trial findings,[52] referencing other reports about the trial that may have a different focus (e.g., process evaluations),[20] and issues related to conflicts of interest (e.g., researcher development of the intervention) and ethical considerations (e.g., informed consent by participants with limited mental capacity).[9]

These reporting standards should be considered through consensus methods, such as a Delphi process and formal consensus meeting.[3], [4] In addition to the standards identified in this review, there may be other factors that have not yet been included in relevant reporting guidelines that could emerge using a rigorous consensus processes. Given the plethora of possible reporting standards, a formal consensus development process would best ensure that new guidance incorporates collective wisdom while providing only the minimal, essential standards for reporting these trials.

### Implications

The CONSORT Statement has been extended and modified in the past, and the CONSORT Group welcomes further extensions.[53] CONSORT guidelines have been developed and validated in the context of biomedical treatments; their applicability to other disciplines could be improved by accounting for specific methodological issues related to the assessment of social and psychological interventions. Members of previous CONSORT groups, journal editors, and researchers believe that stakeholders need to be included in guideline development to promote buy-in and to improve the relevance of CONSORT guidelines to disciplines outside medicine.[12], [54] This review demonstrates that a unified set of standards could be applied to social and psychological intervention trials. Moreover, the impact of CONSORT and the recent proliferation of publications about reporting quality in social and behavioural sciences indicate that such a CONSORT extension could be well-received by various stakeholders.

Since the conduct of this review, an international collaboration of stakeholders has convened to develop a new CONSORT extension for social and psychological interventions. This CONSORT extension has the potential to benefit this area of research in several ways. Developed and disseminated according to recommended techniques,[3] it will aim to synthesise previous work on reporting standards and methodological research about social and psychological interventions. This guideline could improve the reporting and utility of these trials for various stakeholders, including trial report authors, systematic reviewers, journal editors, peer-reviewers, funding organisations, research students, and users of research in policy and practice. While trials are not the only method for evaluating interventions, nor are they the only method that can benefit from updated reporting standards,[50] the importance of trial reports is growing. Improved reporting is needed so that judgments can be made about the validity and application of research findings.[41] A CONSORT extension for social and psychological interventions would be an important step towards improving the reporting of these trials.

## Supporting Information

Appendix S1 Social and psychological intervention RCT reporting standard coding sheet.
(DOC)

Appendix S2 Data extraction sheet for reporting guidelines and quality assessment tools
(DOC)

Table S1 Reported details of guideline development methods.
(DOC)

Table S2 New and modified reporting standards for social and psychological intervention RCTs.
(DOC)

Table S3 Frequency of compliance with reporting standards.
(DOC)

Text S1 Electronic search strategy.
(DOC)

Data File S1 Excel file of RCT reporting quality data.
(XLSX)
