# Supplementary material for: Reporting Quality of Social and Psychological Intervention Trials: A Systematic Review of Reporting Guidelines and Trial Publications
Source: PLoS One. 2013 May 29;8(5):e65442. doi: 10.1371/journal.pone.0065442 (PMC3666983; doi:10.1371/journal.pone.0065442)
Supplement: Appendix S1 — Social and psychological intervention RCT reporting standard coding sheet. (DOC) [file pone.0065442.s001.doc]

**Appendix A. Social and psychological intervention RCT reporting standard coding sheet**

| **Reviewer name:** |
| --- |
| **Date of coding:** |
| **First author of paper:** |
| **Year of publication:** |
| **Journal of publication:** |
| **Social Science Discipline:** |

**Derived from:**

1. American Education Research Association Standards (AERA 2006)

2.   CONSORT Abstracts Extension (Hopewell 2008)

3.   CONSORT Alcohol Outcome Studies Coding Sheet (Ladd 2010)

4.   CONSORT Cluster Trials Extension (Campbell 2004)

5. CONSORT Criminal Justice Trials Coding Sheet (Perry 2008)

6.   CONSORT Harms Extension (Ioannidis 2004)

7.   CONSORT Non-Pharmacological Extension (Boutron 2008)

8.   CONSORT Pragmatic Trials Extension (Zwarenstein 2008)

9. CONSORT Statement (Schulz 2010)

10. Evidence-Based Behavioral Medicine Guidelines (Davidson 2003)

11. Jadad Scale (Jadad 1996)

12. Journal Article Reporting Standards (APA 2008)

13. Nelson-Moberg Expanded CONSORT Instrument (Nelson 2004)

14. Oxford Implementation Index (Montgomery, cited in Eisentstein 2007)

15. Quality Evaluation Form (Balas 1995)

16. Reporting Standards for Clinical Trials (Mosteller 1980)

17. TREND Statement (Des Jarlais 2004)

18. Standards of Reporting Trials Group Recommendations (SRTG 1994)

19. WIDER Recommendations (Abraham 2009)

*Indicates a new or modified standard to official CONSORT guidance

| **Reporting Item: Did the paper report?** | **Section or Topic** | **Guidance for reviewer assessment** | **Score (Yes = 1, No = 0)** |
| --- | --- | --- | --- |
|
| 1a. Identification as randomised trial in title | Title/Abstract | The authors describe the trial as having a randomised design in the title |  |
| 1b. Identification as randomised trial in abstract | Title/Abstract | The authors describe the trial as having a randomised design in the abstract |  |
|
| 1c. Participants | Title/Abstract | The authors report primary eligibility criteria for participants in the abstract |  |
| 1d. Setting | Title/Abstract | The authors report the particular setting(s) in the abstract |  |
| 1e. Intervention group(s) | Title/Abstract | The authors report the treatment(s) intended for each intervention group in the abstract |  |
| 1f. Control | Title/Abstract | The authors report the treatment(s) intended for control in the abstract |  |
| 1g. Care provider | Title/Abstract | The authors give information on who provided the treatments in the abstract |  |
| 1h. Objective | Title/Abstract | The authors report a specific objective or hypothesis in the abstract |  |
| 1i. Primary outcome | Title/Abstract | The authors report a clearly defined primary outcome for this report in the abstract |  |
| 1j. Blinding participants | Title/Abstract | The authors report whether or not participants were blinded to group assignment in the abstract |  |
| 1k. Blinding providers | Title/Abstract | The authors report whether or not providers were blinded to group assignment in the abstract |  |
| 1l. Blinding outcome assessors | Title/Abstract | The authors report whether or not those assessing the outcomes were blinded to group assignment in the abstract |  |
| 1m. Numbers randomised | Title/Abstract | The authors list the number of participants randomised to each group in the abstract |  |
| 1n. Numbers analysed | Title/Abstract | The authors list the number of participants analysed in each group for the primary outcome in the abstract |  |
| 1o. Result for primary outcome | Title/Abstract | For the primary outcome, the authors provide a result for each group, including the estimated effect size and its precision in the abstract |  |
| 1p. Conclusion | Title/Abstract | The authors describe general interpretation of the results in the abstract |  |
| 2a. Background: condition* | Introduction | The authors report the importance of the condition (e.g., nature of the problem, prevalence and incidence, course without treatment) |  |
| 2b. Background: intervention* | Introduction | The authors report background research about the intervention to be evaluated (e.g., intervention development, previous effectiveness studies) |  |
| 2c. Background: theory of change* | Introduction | The authors report the assumed theory of change underlying intervention |  |
| 2d. Rationale | Introduction | The authors present clear reasons for conducting the study (e.g., how the study will add to knowledge in field or address gap in research) |  |
| 2e. Objectives | Introduction | The authors present specific objectives of the study (i.e., aims of the study) |  |
| 2f. Hypotheses* | Introduction | The authors present specific hypotheses of the study (e.g., research questions of the study, expected findings) |  |
| 3a. Trial Design | Methods | The authors report the nature of the randomised trial design (e.g., parallel, factorial, cluster) |  |
| 3b. Allocation ratio | Methods | The authors explicitly report the allocation ratio of the trial |  |
| 3c. Protocol deviations | Methods | The authors report whether there were any important changes to methods after trial commencement (such as eligibility criteria), and reasons if so |  |
| 4a. Eligibility criteria | Methods | The authors clearly report all inclusion and exclusion criteria for the participants (Note: a partial score of .5 for reporting some criteria but not list inclusion and exclusion criteria or explicitly stating that “this is all the criteria”: need that statement for a 1) |  |
| 4b. Concurrent secular events* | Methods | The authors report relevant external events occurring at the time of intervention that could influence outcomes (e.g., media campaigns, political movements, demographic changes, climatic events) |  |
| 4c. Setting type* | Methods | The authors state the type of settings of data collection in the study (e.g., classroom, participants’ homes) |  |
| 4d. Setting number* | Methods | The authors state the number of settings of data collection in the study (i.e., number of classrooms, participants’ homes) |  |
| 4e. Location* | Methods | The authors describe the geographical area in which the study occurred (e.g., city, country, region) |  |
| 4f. Timing* | Methods | The authors state the time period of data collection (e.g., month and year) |  |
| 4g. Patient preference* | Methods | The authors provide information about treatment preferences of participants |  |
| 4h. Provider preference* | Methods | The authors provide information about treatment preferences of providers |  |
| 5a. Service environment characteristics* | Methods | The authors provide characteristics of the service environment (e.g., economic, legal, political, demographic, technological, and policy-related environment; availability of alternatives outside the trial context; compensation structures; unique features of the trial environment) |  |
| 5b. Delivering organisation characteristics* | Methods | The authors provide characteristics of the delivering organisation (e.g., resources, programme advocates, interagency links, management, organisational compatibility with the intervention) |  |
| 5c. Design: intervention treatment | Methods | The authors clearly state the intended treatment for the intervention group(s) (e.g., what it is, core components of treatment, and sequence of activities) |  |
| 5d. Design: control treatment | Methods | The authors clearly state the intended treatment for the control group(s) (e.g., what it is, core components of treatment and sequence of activities) |  |
| 5e. Design: proscribed intervention components* | Methods | The authors describe activities or other elements proscribed in intervention group (i.e., any activities or components that are incompatible with the treatment) |  |
| 5f. Design: proscribed control components* | Methods | The authors describe activities or other elements proscribed in control group (i.e., any activities or components that are incompatible with the treatment) |  |
| 5g. Design: intervention materials* | Methods | The authors describe any necessary materials, technology, and/or technical requirements for standardised treatment |  |
| 5h. Design: control materials* | Methods | The authors describe any necessary materials, technology, and/or technical requirements for standardised treatment |  |
| 5i. Design: intervention format | Methods | The authors state the intended format of session and method of delivery of intervention (e.g., group or individual sessions, internet or face-to-face) |  |
| 5j. Design: control format | Methods | The authors state the intended format of session and method of delivery of control (e.g., group or individual sessions, internet or face-to-face) |  |
| 5k. Design: intervention duration | Methods | The authors state the intended length of the intervention (e.g., 20 week programme) |  |
| 5l. Design: control duration | Methods | The authors state the intended length of control (e.g., 20 week programme) |  |
| 5m. Design: intervention frequency | Methods | The authors state the frequency of intervention (e.g., 1 per week) |  |
| 5n. Design: control frequency | Methods | The authors state the frequency of control (e.g., 1 per week) |  |
| 5o. Design: intervention intensity | Methods | The authors state the intended intensity of the intervention (i.e., length of each session) |  |
| 5p. Design: control intensity | Methods | The authors state the intended intensity of the control (i.e., length of each session) |  |
| 5q. Design: intervention staffing | Methods | The authors state the intended staffing of the intervention (e.g., training and qualifications) |  |
| 5r. Design: control staffing | Methods | The authors state the intended staffing of the control (e.g., training and qualifications) |  |
| 5s. Delivery: intervention treatment* | Methods | The authors clearly state the delivered treatment for the intervention group(s) (i.e., actual activities delivered) |  |
| 5t. Delivery: control treatment* | Methods | The authors clearly state the delivered treatment for the control group(s) (i.e., actual activities delivered) |  |
| 5u. Delivery: programme differentiation* | Methods | The authors describe differences between or within trial arms in treatment delivery by staff |  |
| 5v. Delivery: proscribed intervention components* | Methods | The authors describe activities or other elements delivered that were proscribed in intervention protocol (i.e., any activities or components that are incompatible with the treatment) |  |
| 5w. Delivery: proscribed control components* | Methods | The authors describe activities or other elements delivered that were proscribed in control protocol (i.e., any activities or components that are incompatible with the treatment) |  |
| 5x. Delivery: non-specific intervention components* | Methods | The authors describe intervention components that are not specifically part of the protocol |  |
| 5y. Delivery: non-specific control components* | Methods | The authors describe control components that are not specifically part of the protocol |  |
| 5z. Delivery: intervention materials* | Methods | The authors describe any necessary materials, technology, and/or technical requirements actually used to deliver intervention as standardised |  |
| 5aa. Delivery: control materials* | Methods | The authors describe any necessary materials, technology, and/or technical requirements actually used to deliver control as standardised |  |
| 5bb. Delivery: intervention format* | Methods | The authors state the actual format of session and method of delivery of intervention (e.g., group and number of people in group, individual sessions, internet or face-to-face) |  |
| 5cc. Delivery: control format* | Methods | The authors state the actual format of session and method of delivery of control (e.g., group and number of people in group, individual sessions, internet or face-to-face) |  |
| 5dd. Delivery: intervention duration | Methods | The authors state the actual length of treatment delivered (e.g., 20 weeks of programming) |  |
| 5ee. Delivery: control duration | Methods | The authors state the actual length of control delivered (e.g., 20 weeks of programming) |  |
| 5ff. Delivery: intervention frequency | Methods | The authors state the actual frequency of treatment delivered (e.g., 1 intervention meeting per week) |  |
| 5gg. Delivery: control frequency | Methods | The authors state the actual frequency of treatment delivered (e.g., 1 control meeting per week) |  |
| 5hh. Delivery: intervention intensity | Methods | The authors state the actual intensity of the intervention delivered (i.e., average length of each session) |  |
| 5ii. Delivery: control intensity | Methods | The authors state the actual intensity of the control delivered (i.e., average length of each session) |  |
| 5jj. Delivery: tailoring of intervention* | Methods | The authors describe other types of intervention adaptation by trialists and staff (e.g., types and extent of deviations from protocol that have not been highlighted above, including supplementing the treatment) |  |
| 5kk. Delivery: tailoring of control* | Methods | The authors describe other types of control adaptation by trialists and staff (e.g., types and extent of deviations from protocol that have not been highlighted above, including supplementing the treatment) |  |
| 5ll. Delivery: intervention staffing | Methods | The authors state the actual staffing of the intervention (i.e., number and qualifications of staff) |  |
| 5mm. Delivery: control staffing | Methods | The authors state the actual staffing of the control (i.e., number and qualifications of staff) |  |
| 5nn. Delivery: intervention provider training* | Methods | The authors state how intervention providers were trained, or that training was not necessary |  |
| 5oo. Delivery: control provider training* | Methods | The authors state how control providers were trained, or that training was not necessary |  |
| 5pp. Delivery: intervention supervision* | Methods | Authors report any contacts between intervention providers and supervisors/researchers |  |
| 5qq. Delivery: control supervision* | Methods | The authors report any contacts between control providers and supervisors/researchers |  |
| 5rr. Delivery: provider adherence measurement* | Methods | The authors describe steps to measure adherence of care providers with the protocol (e.g., incentives for staff compliance, participant feedback, monthly mailings or weekly phone reminders) |  |
| 5ss. Delivery: participant compliance measurement* | Methods | The authors describe steps to measure compliance of participants with the protocol (e.g., steps to reduce contamination between trial arms, monthly mailings or weekly phone reminders, any efforts to discourage uptake of interventions outside the trial context) |  |
| 5tt. Uptake: intervention treatment* | Methods | The authors describe the treatment taken up by intervention group(s) participants (i.e., compliance to core components of treatment and sequence of activities) |  |
| 5uu. Uptake: control treatment* | Methods | The authors describe the treatment taken up by control group(s) participants (i.e., compliance to components of treatment and sequence of activities) |  |
| 5vv. Uptake: programme differentiation* | Methods | The authors describe differences between or within trial arms in treatment uptake by participants |  |
| 5ww. Uptake: contamination of intervention* | Methods | The authors describe participant uptake of treatments outside the intervention protocol (includes uptake of control group treatment) |  |
| 5xx. Uptake: contamination of control* | Methods | The authors describe participant uptake of treatments outside the control protocol (includes uptake of intervention group treatment) |  |
| 5yy. Uptake: proscribed intervention components* | Methods | The authors describe activities or other elements sought by participants that were proscribed in intervention protocol, or report that this data was not recorded (i.e., any activities or components that are incompatible with the treatment) |  |
| 5zz. Uptake: proscribed control components* | Methods | The authors describe activities or other elements sought by participants that were proscribed in control protocol (i.e., any activities or components that are incompatible with the treatment) |  |
| 5aaa. Uptake: intervention materials* | Methods | The authors describe any necessary materials, technology, and/or technical requirements actually used by participants in intervention group |  |
| 5bbb. Uptake: control materials* | Methods | The authors describe any necessary materials, technology, and/or technical requirements actually used by participants in control group |  |
| 5ccc. Uptake: intervention frequency* | Methods | The authors state the actual number of treatment sessions utilised by intervention participants (e.g., 16 weeks of 20 week treatment) |  |
| 5ddd. Uptake: control frequency* | Methods | The authors state the actual number of treatment sessions utilised by control participants (e.g., 16 weeks of 20 week treatment) |  |
| 5eee. Uptake: intervention intensity* | Methods | The authors state the actual intensity of the intervention utilised by intervention participants (i.e., average length of each session) |  |
| 5fff. Uptake: control intensity* | Methods | The authors state the actual intensity of the control utilised by control participants (i.e., average length of each session) |  |
| 5ggg. Uptake: enactment* | Methods | The authors report on the extent to which participants implement specific treatment-related activities (e.g., learned behavioural skills and cognitive strategies) in relevant real-life settings |  |
| 6a. Baseline data collection | Methods | The authors describe the conditions for baseline data collection (e.g., setting of data collection, method of data collection) |  |
| 6b. Baseline data measures | Methods | The authors describe the measures used for baseline data collection |  |
| 6c. Primary outcome data collection | Methods | The authors report how they measured the primary outcome (e.g., setting of data collection, method of data collection) |  |
| 6d. Primary outcome measures* | Methods | The authors clearly identify and describe the primary outcome measure(s), two at most (e.g., whether outcome measures were validated, pre-existing, or developed by authors themselves) |  |
| 6e. Secondary outcome data collection* | Methods | The authors report how they measured any secondary outcome(s) (e.g., setting of data collection, method of data collection) |  |
| 6f. Secondary outcome measures* | Methods | The authors describe all of the secondary outcome measures (e.g., whether outcome measures were validated, pre-existing, or developed by authors themselves) |  |
| 6g. Period of follow-up | Methods | The authors clearly state the follow-up period for all groups and measures in the trial (i.e., fixed follow-up period or saturation point of data) |  |
| 6h. Methods to enhance quality of measurements* | Methods | The authors report methods to improve the quality of data measurements (e.g., double scoring of questionnaires, inter-rater reliability of data collection/data entry procedures, training requirements, and coded audio/visual tapes) |  |
| 6i. Changes to data collection protocol* | Methods | The authors report whether there were any changes to the protocol for measuring and analysing trial outcomes after the trial commenced, with reasons |  |
| 7a. Sample size calculation | Methods | The authors provide clear information and justification for sample size calculation |  |
| 7b. Interim analyses and stopping rules | Methods | The authors clearly state whether they planned to collect interim results and under what circumstances they would have stopped the trial |  |
| 8a. Random Sequence Generation | Methods | The authors clearly state the method of random allocation of participants (e.g., computer generated sequences, random numbers table, coin toss) |  |
| 8b. Randomisation restrictions | Methods | The authors describe details of any restrictions (e.g. blocking, ratios, and explanation of why restrictions employed) |  |
| 8c. Allocation of providers* | Methods | The authors clearly state the method of allocation of care providers |  |
| 9. Allocation concealment | Methods | The authors describe methods used to access, conceal, and implement the random allocation sequence (e.g., use of the telephone or reading numbers off a list) |  |
| 10a. Sequencer Generator | Methods | The authors clearly state the person who generated the allocation sequence |  |
| 10b. Who enrolled participants | Methods | The authors clearly state who enrolled the participants |  |
| 10c. Who assigned participants | Methods | The authors clearly state who assigned the participants to the groups |  |
| 11a. Provider blinding* | Methods | The authors clearly report if and how treatment providers were blinded to group assignment, or if not, why |  |
| 11b. Participant blinding* | Methods | The authors clearly report if and how participants were blinded to group assignment, or if not, why |  |
| 11c. Assessor blinding | Methods | The authors clearly report if and how outcome assessors were blinded to group assignment, or if not, why |  |
| 12a. Primary outcome analytic plan* | Methods | The authors clearly state the pre-determined data analysis plan for the primary outcome |  |
| 12b. Secondary outcome analytic plan* | Methods | The authors clearly state the pre-determined data analysis plan for the secondary outcome(s) |  |
| 12c. Sub-group and adjusted analyses* | Methods | The authors report the methods of any sub-group and/or adjusted analyses they performed and which were pre-specified |  |
| 13a. Participant flow | Results | The authors clearly show the participants in the trial through use of a flow diagram (from participants approached to numbers in analysis) |  |
| 13b. Number approached | Results | The authors clearly report the number of potential participants approached for eligibility |  |
| 13c. Number eligible | Results | The authors clearly report the number of participants eligible for trial and reasons for non-participation |  |
| 13d. Number randomised | Results | The authors clearly report how many people were allocated to each arm of the trial |  |
| 13e. Treatment allocation | Results | The authors clearly report the number of participants who completed treatment as allocated, by study group |  |
| 13f. Attrition | Results | The authors clearly report the amount of and reasons for participants dropping out of the trial for each trial arm |  |
| 13g. Discontinued Intervention | Results | The authors clearly report the number of participants who discontinued the intervention |  |
| 13h. Number in primary analysis | Results | The authors clearly report the final numbers used in primary analysis for each group |  |
| 14a. Period of recruitment | Results | The authors clearly state the period of recruitment |  |
| 14b. Recruitment process | Results | The authors describe how the participants are approached and selected (have a partial score of .5 for reporting only some criteria) |  |
| 14c. Incentives* | Results | The authors report any incentives or compensation for participating in the trial |  |
| 14d. Reasons for stopping | Results | The authors clearly state why the trial ended or stopped |  |
| 15. Baseline data* | Results | The authors report the demographic details of both groups (e.g., age, ethnicity, gender, socioeconomics, cultural, linguistic, religious, and other relevant characteristics, education, employment, marital status, number taking medication or receiving other therapy) |  |
| 16a. Number analysed | Results | For each group, the authors report the number of participants (denominator) included in each analysis |  |
| 16b. Intention-to-treat* | Results | The authors clearly state precise procedures for intention-to-treat or per-protocol analysis (i.e., not just "Intention-to-treat," but actual plan for dealing with missing data) |  |
| 17a. Primary outcome results* | Results | The authors clearly present all results for primary outcome for each group to allow replication or alternative analyses (e.g., all time intervals, exact P values, effect size, precision) |  |
| 17b. Secondary outcome results* | Results | The authors clearly present all results for secondary outcome(s) for each group to allow replication or alternative analyses (e.g., all time intervals, exact P values, effect size, precision) |  |
| 18. Sub-group or adjusted analysis results | Results | The authors clearly report any other subgroup/adjusted analyses performed |  |
| 19. Adverse events* | Results | The authors describe any adverse events or harms experienced by participants in trial |  |
| 20. Limitations* | Discussion | The authors clearly state any methodological weaknesses or limitations of the study that can lead to imprecision (e.g., dangers associated with multiplicity outcomes, choice of the comparator, lack of or partial blinding, and unequal expertise of care providers or centres in each group) |  |
| 21. Generalisability* | Discussion | The authors clearly state the generalisability of the study according to the intervention as implemented, comparators, patients, care providers and centres involved in the trial, and possible differences to other settings |  |
| 22a. Overall evidence* | Discussion | The authors clearly state how the current findings fit in with the weight of evidence across other trials and studies |  |
| 22b. Results compared to hypotheses* | Discussion | The authors clearly compare the results to predictions made prior to the start of the trial |  |
| 22c. Reference to systematic review | Other Information | The authors made reference to systematic review on subject and how this paper fits, or whether no such paper exists |  |
| 22d. Reference to other studies* | Other Information | The authors indicate whether there are other papers and/or reports on this study and, if so, refers the reader to these papers |  |
| 23. Trial registration | Other Information | The authors provide the registration number and name of trial register |  |
| 24a. Protocol* | Other Information | The authors report where a full trial protocol can be accessed, if available |  |
| 24b. Access to treatment manual* | Other Information | The authors provide information for accessing intervention materials (e.g., protocol or manual) to allow for replication |  |
| 25a. Conflicts of interest* | Other Information | The authors report sources of funding and other support (such as supply of drugs), role of funders, or other biases they researchers have that may influenced their research |  |
| 25b. Ethical considerations* | Other Information | The authors report any ethical considerations for the study (e.g., the study received ethical approval, informed consent) |  |
| 25c. Intervention development* | Other Information | The authors report whether or not they developed the intervention |  |

**Notes on coding:**

Each reporting standard is assigned a “yes” (score of 1) or “no” (score of 0) response depending on whether the authors reported that item. Coding rules were adapted from previous studies about reporting quality. Firstly, each sub-item aims to address a single reporting standard that can be scored by a reviewer who is not an expert in the field. Secondly, one sentence in a trial report that contains multiple pieces of information can achieve compliance to several reporting standards simultaneously. In contrast to previous studies, if no primary outcomes were specified, all outcomes in a trial report are considered secondary outcomes, and all items referring to primary outcomes are coded as 0. Not penalising would under-emphasise the importance of specifying primary outcomes for testing and delineating theory, in addition to their role in power calculations. Lastly, unless specifically required by a reporting item, credit is granted if the authors provide the information in a different section of the paper than is listed in the CONSORT checklist. For example, Items 1a-1p must be reported in the title or abstract, but information on allocation could be in the methods, results, or discussion section.
