# Supplementary material for: Reporting Quality of Social and Psychological Intervention Trials: A Systematic Review of Reporting Guidelines and Trial Publications
Source: PLoS One. 2013 May 29;8(5):e65442. doi: 10.1371/journal.pone.0065442 (PMC3666983; doi:10.1371/journal.pone.0065442)
Supplement: Appendix S2 — Data extraction sheet for reporting guidelines and quality assessment tools (DOC) [file pone.0065442.s002.doc]

**Appendix B. Data extraction sheet for reporting guidelines and quality assessment tools**

**(Modified from Moher 2010).**

| **Domain** | **Item** | **Guidance** | **Response** |
| --- | --- | --- | --- |
| **Descriptives** | Guideline | Title of Guideline |  |
|  | Developer | Name of corresponding author or group in charge of guideline development |  |
|  | Social/Behavioural Science Specific? | Is the reporting guideline for a specific field in social/behavioural science? |  |
|  | Guideline or Quality Measurement? | Is the tool a reporting guideline or a reporting quality assessment tool? |  |
|  | CONSORT | Is it a CONSORT Statement or official extension? |  |
|  | Targeted Area | Targeted research method/area of the guideline? |  |
|  | Number of Reporting Standards | How many reporting standards were included in the guideline? |  |
| **Preliminary Stages** | Poor Reporting | Did the developers empirically demonstrate the need for new guidance, extending existing guidance, or implementing existing guidance? |  |
|  | Previous Standards | Did the developers describe whether they identified previous relevant standards and/or identified key information related to the potential sources of bias in such studies? |  |
|  | Funding | Did the developers report whether they obtained any funding for the reporting standards initiative? |  |
|  | Participants | Did the developers report any participants included in development process? |  |
| **Reporting Standards Document Development** | Delphi Exercise | Did the authors report conducting a Delphi Exercise? |  |
|  | Preliminary Items | Did the developers report whether they generated a preliminary list of items to consider prior to the final selection process? |  |
|  | Finalisation Preparation | Did the authors describe any preparations that developers made prior to the final selection process (e.g., decide size and duration of the face-to-face meeting, develop meeting logistics, develop meeting agenda, consider presentations on relevant background topics, including summary of evidence, plan to share results of Delphi exercise if done, invite session chairs, prepare materials to be sent to participants prior to meeting, arrange to record the meeting)? |  |
|  | Finalisation Process | Did the authors describe the process of reaching consensus on guideline content? (e.g., present and discuss results of pre-meeting activities and relevant evidence at consensus meeting, discuss the rationale for including items in the checklist, discuss the development of a flow diagram, discuss strategy for producing documents, identify who will be involved in which activities, discuss authorship, discuss knowledge translation strategy) |  |
|  | Tool Development | Did the authors describe how the guideline or assessment tool was written? (e.g., pilot testing the checklist, sending drafts electronically to collaborators) |  |
|  | Explanatory Document | Did the developers construct an explanatory document as well? |  |
| **Publication** | Publication Strategy | Did the developers describe a publication strategy, if any? |  |
|  | Criticism and Feedback | Did the developers describe how they planned to deal with criticism and feedback? |  |
|  | Updates | Did the developers describe any plans and/or processes for updates? |  |
| **Dissemination** | Endorsement | Did the developers report any endorsement attained? |  |
|  | Adherence | Did the developers report any processes for seeking adherence to the guideline? |  |
|  | Impact | Did the authors describe a process for evaluating the impact of the guideline or assessment tool, if any? |  |
|  | Website | Is the reporting standards document hosted on an open-access website? |  |
|  | Translation | Is the reporting standards document translated into any other languages? |  |
|  | Citation | Number of citations in Google Scholar of the reporting standards document (and explanatory document, if applicable) |  |
