# Supplementary material for: Reporting Quality of Social and Psychological Intervention Trials: A Systematic Review of Reporting Guidelines and Trial Publications
Source: PLoS One. 2013 May 29;8(5):e65442. doi: 10.1371/journal.pone.0065442 (PMC3666983; doi:10.1371/journal.pone.0065442)
Supplement: Table S1 — Reported details of guideline development methods. (DOC) [file pone.0065442.s003.doc]

**Table S1. Reported details of guideline development methods**

| **Guideline** | **Preliminary Stages** | **Document Development** | **Publication** | **Dissemination** |
| --- | --- | --- | --- | --- |
| Alcohol Outcome Studies Coding Sheet | - Evidence of poor reporting - Identified previous standards - Funded by CASAA - 4 reported participants | - Preliminary list of items generated from 2001 CONSORT Statement - Developed tool by pilot testing and modifying items to fit psychosocial interventions | - No techniques reported | - No techniques reported |
| AERA Standards for Empirical Social Science Research | - 10 reported participants | - No techniques reported | - No techniques reported | - Endorsement by AERA - Available on open-access website |
| CONSORT and Criminal Justice Trials (CJT) Project Coding Sheet | - Evidence of poor reporting - Identified previous standards - Funded by government and university support | - Developed tool via 3-stage process of combining CONSORT items with items from crime and justice literature | - Reported desire to update by developing a CONSORT extension in criminology | - Available on open-access website |
| CONSORT Extension for Abstracts | - Evidence of poor reporting - Identified previous standards - Various funding sources reported - 63 reported participants in Delphi; 26 reported participants in consensus meeting | - Conducted Delphi exercise - Preliminary list of items from review of previous standards - Prepared for finalisation by distributing Delphi results prior to consensus meeting - Finanlisation process via consensus meeting - Developed tool via steering group - Produced E&E document | - Feedback on limitations via CONSORT website - Abstract Extensions as warranted | - Endorsement attained by journals - Adherence by journal editors and conference organisers for submissions - Conducted a review on the impact of the guideline - Available on open-access website |
| CONSORT Extension for Cluster Trials | - Evidence of poor reporting - Identified previous standards - Various funding sources reported | - Finalisation process via face-to-face meetings and teleconferences - Developed via CONSORT Group | - Updated in 2012 | - Endorsement attained by journals - Adherence by journal editors for submissions - Conducted a review on the impact of the guideline - Available on open-access website |
| CONSORT Extension for Non-Pharmacological Treatments | - Evidence of poor reporting - Identified previous standards - Various funding sources reported - 33 reported participants | - Conducted web-based survey of participants to identify potential items - Preliminary list of items from review of previous standards - Prepared for finalisation by distributing survey results prior to consensus meeting - Finanlisation process via consensus meeting - Developed tool via steering committee - Produced E&E document | - Discussed publication of E&E in another journal as part of publication strategy - Invited feedback and will continue to search literature - Plan to develop Non-Pharmacological Extensions | - Endorsement attained by journals - Adherence by journal editors for submissions - Conducted a review on the impact of the guideline - Available on open-access website |
| CONSORT Extension for Pragmatic Trials | - Identified previous standards - Various funding sources reported - 24 reported participants in 2005; 42 in 2008 | - Prepared for finalisation by drafting and distributing a summary paper based on the initial 2005 meeting - Finalisation process via consensus meeting - Developed tool via CONSORT Group | - Submitted to one journal for publication after the consensus meeting | - Endorsement attained by journals - Adherence by journal editors for submissions - Available on open-access website |
| CONSORT Extension for Reporting Harms | - Evidence of poor reporting - Identified previous standards - Various funding sources reported - 31 reported participants | - Preliminary items identified through literature search - Prepared for finalisation by sharing results of literature search with participants - Finalisation process via consensus meeting - Developed tool via circulation of drafts amongst team members | - Invited feedback on guideline website - Update as needed | - Endorsement attained by journals - Adherence by journal editors for submissions - Available on open-access website |
| CONSORT Statement | - Evidence of poor reporting - Identified previous standards - Various funding sources reported - Dozens of participants throughout its 3 iterations | - Conducted Delphi exercise - Preliminary items from literature review and Delphi - Prepared for finalisation by having participants aggregate and share data on potential items - Finalisation process via consensus meeting - Tool developed via CONSORT Executive - Produced E&E document | - Multiple, peer-reviewed publications - Invited feedback on guideline website and in research literature - Updated twice | - Endorsement attained by journals - Adherence by journal editors for submissions - Multiple reviews on its impact - Available on open-access website - Translated into several languages |
| Evidence-Based Behavioral Medicine-Specific Guidelines | - Evidence of poor reporting - Identified previous standards - Funded by NIH OBSSR contract - 9 reported participants | - No techniques reported | - No techniques reported | - No techniques reported |
| Jadad Scale | - Reviewed literature to identify possible standards - 6 participants and 7 authors reported | - Conducted modified nominal group technique - Developed a preliminary list of items to discuss - Prepared for finalisation by voting on face validity of items - Finalisation process via panel of judges pre-testing the draft instrument - Tool developed via panel of judges | - No techniques reported | - No techniques reported |
| Journal Article Reporting Standards | - Evidence of poor reporting - Identified previous standards - Developed by JARS Group, the APA Council of Editors, the APA Publication Manual Revision Task Force, and the Publications and Communications Board. | - Preliminary items were identified from previous guidelines - Prepared for finalisation by sharing draft guideline with various APA groups - Finalisation process via meeting of JARS Group - Developed tool via JARS Group with feedback from various APA groups - Produced E&E document | - Plan to update with additional modules | - Endorsement attained by APA journals - Adherence required by APA journal editors - Checklist is available on an open-access website (E&E document is not) |
| Nelson-Moberg Expanded CONSORT Instrument | - Identified previous standards - 2 reported participants | - Finalisation process via by pilot-testing on 3 articles - Developed tool via refinement after pilot testing - Produced E&E document | - Invite feedback from RCT experts | - No techniques reported |
| Oxford Implementation Index | - Identified previous standards - Various funding sources reported - Unspecified amount of participants at various meetings | - Conducted Delphi exercise - Preliminary items from literature review and Delphi - Prepared for finalisation by sharing results from literature reviews - Finalisation process via consensus meeting - Tool developed via project team - Produced guidance for each item | - No techniques reported | - Available on open-access website |
| Quality Evaluation Form | - Identified previous standards - Funded by government and university support | - No techniques reported | - No techniques reported | - No techniques reported |
| Reporting Standards for Controlled Trials | - Evidence of poor reporting - Funded by government grants | - No techniques reported | - No techniques reported | - Adherence by editor of submitted journal |
| Structured Reporting of Randomized Controlled Trials | - Evidence of poor reporting - Identified previous standards - Various funding sources reported - 30 participants reported | - Conducted a 144-item survey - Generated a list of preliminary items - Prepared for finalisation by organising participants into groups with different focus areas - Finalisation process via presentation of small group recommendations - Tool developed by whole group | - Update via feedback from journal editors and Cochrane groups | - Adherence by journal editors |
| TREND Statement | - Evidence of poor reporting - Identified previous standards - Funded by the CDC - 18 reported participants | - Prepared for finalisation by reviewing literature - Finalisation process via consensus meeting | - Invited feedback - Plan to periodically revise TREND accordingly | - Endorsed by many journals - Adherence during journal submissions - Review done on impact of guideline - Available on open-access website |
| WIDER | - Identified previous standards - 32 reported participants | - No techniques reported | - No techniques reported | - Endorsed by several journal editors - Adherence strategies in journal editorial policy - Available on open-access website |
