# Supplementary material for: Reporting Quality of Social and Psychological Intervention Trials: A Systematic Review of Reporting Guidelines and Trial Publications
Source: PLoS One. 2013 May 29;8(5):e65442. doi: 10.1371/journal.pone.0065442 (PMC3666983; doi:10.1371/journal.pone.0065442)
Supplement: Table S2 — New and modified reporting standards for social and psychological intervention RCTs. (DOC) [file pone.0065442.s004.doc]

**Table S2. New and modified reporting standards for social and psychological intervention RCTs**

| **Reporting Standards Document** | **New/Modified CONSORT Standard** | **Elongated CONSORT Checklist Item(s)** |
| --- | --- | --- |
| **Alcohol Outcome Studies Coding Sheet** |  |  |
|  | Report appropriate quantitative measures of sample and target population to discuss external validity | 21. Generalisability |
| **AERA Standards for Empirical Social Science Research** |  |  |
|  | Report the historical context of the phenomena studied | 5a. Service Environment Characteristics |
|  | Report how the measurement or classification of latent constructs preserves important characteristics of the phenomena under study and is relevant to capturing important characteristics of the group studied, either by referencing a publication on the measure or describing measurement development | 6d. Primary outcome measures  6f. Secondary outcome measures |
|  | Provide a sufficient description of development, guides, protocols, and context of qualitative process evaluations (e.g., interviews, open-ended surveys, observational inventories) | 6e. Secondary outcome data collection |
|  | Report the process of analysing and interpreting claims from qualitative process evaluations (e.g., practices used to develop and enhance the warrant for the claims, including the search for disconfirming evidence and alternative interpretations of the same evidence) | 12b. Secondary outcome analytic plan |
|  | Present the evidence that serves as a warrant for each claim from qualitative evaluations | 17b. Secondary outcome results |
|  | Delineate the situations (or domains) in which the findings of the investigation do not apply to identify the scope of intended generalisation | 21. Generalisability |
|  | Describe how and why the patterns of qualitative responses may have occurred (e.g., the social, cultural, or historical contexts, how they relate to one another, how they support or challenge theory and findings from previous research, and what alternative claims or counter-claims were considered) | 22a. Overall evidence |
|  | Include a description of any personal biases of the researcher that may have influenced or could have the appearance of influencing the research, along with a description of how they were managed in the conduct of the study | 25a. Conflicts of interest |
|  | (1) Describe ethical considerations involved in data collection, analysis, and reporting; (2) Report whether IRB approval was obtained; (3) Report research and findings in a way that honours consent agreements with human participants and any other agreements with respect to gaining access to research sties or data | 25b. Ethical approval |
| **CONSORT Criminal Justice Trials Project Coding Sheet** |  |  |
|  | Report any measures indicating quality of latent construct measurement, such as double scoring of questionnaires, inter-rater reliability of data collection/data entry procedures, training requirements, protocol adherence, coded audio/visual tapes, supervision and feedback of staff. | 6h. Methods to enhance quality of measurements |
|  | Report on the background characteristics of the groups, including criminal history, targeted criminogenic needs, specifically targeted offence types, and family history | 15. Baseline data |
|  | Clearly state any social harm done to participants (e.g., increased criminal activity following the interventions) | 19. Adverse events |
|  | (1) Report whether other paper(s) on the study exist; (2) Report whether this paper represents a set of experiments | 22d. Reference to other papers on this study |
|  | Report whether the authors developed and evaluated the intervention | 25c. Intervention development |
| **Evidence-Based Behavioral Medicine-Specific Guidelines** |  |  |
|  | Describe health/social outcome in sufficient detail to allow the reader to consider secular trends | 4b. Concurrent secular events |
|  | Report the treatment preference of the participants | 4g. Participant preference |
|  | Report the treatment preference of the providers | 4h. Provider preference |
|  | Report the success of treatment integrity by the provider | 5s. Delivery: intervention treatment  5t. Delivery: control treatment |
|  | Report the specific procedures that were used to train providers to uniformly conduct the study treatments | 5nn. Delivery: intervention provider training  5oo. Delivery: control provider training |
|  | Report the type, duration, and form that supervision, if any, required | 5pp. Delivery: intervention supervision  5qq. Delivery: control supervision |
|  | Report adherence measures and the decision rules, if any, whereby these adherence measures were combined | 5ss. Delivery: participant compliance measurement |
|  | Report treatment adherence of participants | 5tt. Uptake: intervention treatment  5uu. Uptake: control treatment |
|  | Report whether participants used intervention materials | 5aaa. Uptake: intervention materials  5bbb. Uptake: control materials |
|  | Report whether an adequate “dose” of the intervention was received | 5ccc. Uptake: intervention frequency  5ddd. Uptake: control frequency  5eee. Uptake: intervention intensity  5fff. Uptake: control intensity |
|  | Report whether or not patients enacted the treatment recommendations outside of intervention sessions | 5ggg. Uptake: enactment |
|  | Report consideration of any measurement demand characteristics of subjective outcome measures | 6h. Methods to enhance quality of measurements |
|  | Report whether any mediation testing was performed, and if so, how | 12b. Secondary outcome analytic plan |
| **Journal Article Reporting Standards (APA)** |  |  |
|  | If other aspects of this study have been reported on previously, describe how the current report differs from these earlier reports | 2b. Background: intervention |
|  | Report the theories or other means used to derive hypotheses | 2f. Hypotheses |
|  | Describe specialised equipment by model and supplier | 5z. Delivery: intervention materials  5aa. Delivery: control materials |
|  | Report whether the intervention involved any language translation, and, if so, the translation method | 5jj. Delivery: tailoring of intervention  5kk. Delivery: tailoring of control |
|  | Report whether there were any estimation problems (e.g., failure to converge, bad solution spaces) or anomalous data points in complex data set | 6i. Changes to data collection protocol |
|  | Report the number of deliverers and, in the case of interventions, the M, SD, and range of number of individuals/units treated by each | 8c. Allocation of providers |
|  | Report empirical evidence and/or theoretical arguments for the causes of data that are missing (i.e., missing completely at random (MCAR), missing at random (MAR), or missing not at random (MNAR)) | 16b. Intention-to-treat |
|  | For multivariable analytic systems (e.g., multivariate analyses of variance, regression analyses, structural equation modeling analyses, and hierarchical linear modeling), also include the associated variance–covariance (or correlation) matrix or matrices | 17a. Primary outcome results 17b. Secondary outcome results  18. Sub-group or adjusted analysis results |
|  | Report whether this data has previously appeared in any dissertations or conference papers | 22d. Reference to other papers on this study |
|  | Report any relationships that may be perceived as conflicts of interest | 25a. Conflicts of interest |
| **Nelson-Moberg Expanded CONSORT Instrument** |  |  |
|  | Describe the problem that necessitated the work – the nature, scope, and severity | 2a. Background: condition |
|  | Report any previous evidence of benefits of active interventions included in trial | 2b. Background: intervention |
|  | (1) Provide a full explication of the theoretical basis of an intervention; (2) Report theory-delineated proximal and distal outcomes | 2c. Background: theory of change |
|  | Differentiate between data collection and trial setting | 4c. Setting type  4d. Setting number  4e. Location |
|  | Report the extent to which participants perceive, comprehend, and use the intervention as intended | 5tt. Uptake: intervention treatment  5uu. Uptake: control treatment |
|  | Report any threats to treatment enactment (e.g., not practicing the behavior, forgetting to do it, being unsure of the correct way to do it, experiencing a lack of success when doing it, lacking a suitable setting, and losing interest in the intervention) | 5ggg. Uptake: enactment |
|  | Given that complex social intervention have a multiplicity of outcomes, report up to 2 primary outcomes | 6d. Primary outcome measures |
|  | Report the use validated latent construct (and their appropriate psychometric properties) or give rationale concerning non-availability or inappropriateness of validated scales (and report the appropriate psychometric properties of scales used) | 6d. Primary outcome measures  6f. Secondary outcome measures |
|  | Report whether the researchers used any methods to compensate for inability to blind providers | 11a. Provider blinding |
|  | Report whether the researchers used any methods to compensate for inability to blind participants | 11b. Participant blinding |
|  | In the protocol, report components that are not part of the intervention and may be part of the competing intervention | 24a. Protocol |
|  | Report whether informed consent was obtained prior to randomisation | 25b. Ethical approval |
| **Oxford Implementation Index** |  |  |
|  | Report any external events at the same time as the trial (e.g., media campaigns, political movements, demographic changes, climatic events) | 4b. Concurrent secular events |
|  | (1) Report the legal, political, demographic, technological, and policy-related environment of the trial; (2) Report the availability of alternatives outside the trial context; (3) Report the compensation structures for interventions; (4) Report unique features of the trial service environment (e.g., free child care, home visits) | 5a. Service environment characteristics |
|  | Report the existence and influence of organisational resources, programme champions, interagency links, management, and organisational compatibility with the intervention | 5b. Delivering organisation characteristics |
|  | Report any activities or components that are incompatible with the treatment | 5e. Design: proscribed intervention components  5f. Design: proscribed control components |
|  | Report any technical requirements (e.g., support staff) | 5g. Design: intervention materials  5h. Design: control materials |
|  | If there are systematic differences between treatments delivered to participants in the same trial arm (e.g., if men and women are treated differently), these should also be noted | 5u. Delivery: programme differentiation |
|  | Report the actual delivery of any activities or components that are incompatible with the treatment | 5v. Delivery: proscribed intervention components  5w. Delivery: proscribed control components |
|  | Report about any treatment components that are not specifically part of the protocol, such as expertise or quality of the therapeutic alliance | 5x. Delivery: non-specific intervention components  5y. Delivery: non-specific control components |
|  | Report the actual use of technical requirements for delivery of materials (e.g., support staff) | 5z. Delivery: intervention materials  5aa. Delivery: control materials |
|  | Report the nature of any contact between staff and trialists/programme developers | 5pp. Delivery: intervention supervision  5qq. Delivery: control supervision |
|  | Report the actual sequence of core steps, stages, or activities that participants took up | 5tt. Uptake: intervention treatment  5uu. Uptake: control treatment |
|  | (1) Report any differences in receipt between active and control arms that have not been highlighted elsewhere; (2) If there are systematic differences between treatments delivered to participants in the same trial arm (e.g., if men and women are treated differently), these should also be noted | 5vv. Uptake: programme differentiation |
|  | Report contamination and uptake of treatments outside the trial context | 5ww. Uptake: contamination of intervention  5xx. Uptake: contamination of control |
|  | Report any activities or components that are incompatible with the treatment | 5yy. Uptake: proscribed intervention components  5zz. Uptake: proscribed control components |
|  | Report participant use of technology, materials, or technical requirements | 5aaa. Uptake: intervention materials  5bbb. Uptake: control materials |
|  | Report socioeconomic, demographic, cultural, linguistic, religious, and other relevant characteristics of participants | 15. Baseline data |
|  | Report any unique ethical considerations found in complex interventions (e.g., parental consent and parental observation when working with children) | 25b. Ethical approval |
| **TREND Statement** |  |  |
|  | Report information on target population | 1c. Participants |
|  | Report how subjects were grouped during delivery | 5bb. Delivery: intervention format  5cc. Delivery: control format |
|  | Report the statistical software if specialised procedures were used | 12a. Primary outcome analytic plan  12b. Secondary outcome analytic plan  12c. Sub-group and adjusted analyses |
|  | Report any activities to increase compliance, adherence, or retention | 14.c Incentives |
|  | (1) Provide comparison between study population at baseline and target population of interest; (2) Provide baseline comparisons of those lost to follow-up and those retained, overall and by study condition | 15. Baseline data |
|  | Report the results from testing prespecified causal pathways through which the intervention was intended to operate, if any | 17b. Secondary outcome results |
|  | Discuss the success of and barriers to implementing the intervention | 20. Limitations |
|  | Report considerations of any incentives, compliance rates, and specific sites/settings involved in the study | 21. Generalisability |
|  | Report programmatic or policy implications of the trial | 22a. Overall evidence |
|  | Discuss results taking into account the mechanism by which the intervention was intended to work (causal pathways) or alternative mechanisms or explanations | 22b. Summary of results compared to objectives/hypotheses |
| **WIDER** |  |  |
|  | Report essential aspects of the intervention development | 2b. Background: intervention |
|  | (1) Report the active change techniques used in the intervention; (2) Report the causal processes targeted by these change techniques | 2c. Background: theory of change |
