# Supplementary material for: Reporting Quality of Social and Psychological Intervention Trials: A Systematic Review of Reporting Guidelines and Trial Publications
Source: PLoS One. 2013 May 29;8(5):e65442. doi: 10.1371/journal.pone.0065442 (PMC3666983; doi:10.1371/journal.pone.0065442)
Supplement: Table S3 — Frequency of compliance with reporting standards. (DOC) [file pone.0065442.s005.doc]

**Table S3. Frequency of compliance with reporting standards**

| **Heading** | **Item** | **Clinical Psychology** | **Criminology** | **Education** | **Social Work** | **Total Sample** |
| --- | --- | --- | --- | --- | --- | --- |
| **Title/Abstract** | 1a. Identification as randomised trial in title | 41.4% | 6.5% | 1.1% | 15.0% | 19.7% |
|  | 1b. Identification as randomised trial in abstract | 81.8% | 22.6% | 31.5% | 75.0% | 54.8% |
|  | 1c. Participants | 87.9% | 32.3% | 65.2% | 95.0% | 72.8% |
|  | 1d. Setting | 22.2% | 0.0% | 30.3% | 40.0% | 23.8% |
|  | 1e. Intervention group(s) | 86.9% | 48.4% | 76.4% | 90.0% | 78.2% |
|  | 1f. Control | 77.8% | 35.5% | 60.7% | 55.0% | 64.0% |
|  | 1g. Care provider | 16.2% | 0.0% | 6.7% | 10.0% | 10.0% |
|  | 1h. Objective | 98.0% | 90.3% | 97.8% | 95.0% | 96.7% |
|  | 1i. Primary outcome | 27.3% | 3.2% | 6.7% | 0.0% | 14.2% |
|  | 1j. Blinding participants | 0.0% | 0.0% | 1.1% | 0.0% | 0.4% |
|  | 1k. Blinding providers | 0.0% | 0.0% | 1.1% | 0.0% | 0.4% |
|  | 1l. Blinding outcome assessors | 5.1% | 0.0% | 1.1% | 0.0% | 2.5% |
|  | 1m. Numbers randomised | 11.1% | 3.2% | 4.5% | 20.0% | 8.4% |
|  | 1n. Numbers analysed | 3.0% | 3.2% | 0.0% | 0.0% | 1.7% |
|  | 1o. Result for primary outcome | 6.1% | 0.0% | 1.1% | 0.0% | 2.9% |
|  | 1p. Conclusion | 88.9% | 41.9% | 68.5% | 60.0% | 72.8% |
| **Introduction** | 2a. Background: condition | 84.8% | 90.3% | 87.6% | 80.0% | 86.2% |
|  | 2b. Background: intervention | 87.9% | 96.8% | 96.6% | 95.0% | 92.9% |
|  | 2c. Background: theory of change | 72.7% | 93.5% | 93.3% | 100.0% | 85.4% |
|  | 2d. Rationale | 96.0% | 96.8% | 91.0% | 95.0% | 94.1% |
|  | 2e. Objectives | 100.0% | 100.0% | 98.9% | 100.0% | 99.6% |
|  | 2f. Hypotheses | 84.8% | 74.2% | 83.1% | 60.0% | 80.8% |
| **Methods** | 3a. Trial Design | 98.0% | 100.0% | 100.0% | 100.0% | 99.2% |
|  | 3b. Allocation ratio | 17.2% | 9.7% | 3.4% | 20.0% | 11.3% |
|  | 3c. Protocol deviations | 6.1% | 9.7% | 6.7% | 25.0% | 8.4% |
|  | 4a. Eligibility criteria | 71.7% | 50.0% | 50.0% | 65.0% | 60.3% |
|  | 4b. Concurrent secular events | 7.1% | 25.8% | 2.2% | 30.0% | 9.6% |
|  | 4c. Setting type | 77.8% | 77.4% | 85.4% | 85.0% | 81.2% |
|  | 4d. Setting number | 65.7% | 54.8% | 66.3% | 85.0% | 66.1% |
|  | 4e. Location | 74.7% | 87.1% | 83.1% | 80.0% | 79.9% |
|  | 4f. Timing | 37.4% | 19.4% | 15.7% | 35.0% | 26.8% |
|  | 4g. Patient preference | 20.2% | 6.5% | 16.9% | 25.0% | 17.6% |
|  | 4h. Provider preference | 2.0% | 0.0% | 3.4% | 5.0% | 2.5% |
|  | 5a. Service environment characteristics | 23.2% | 45.2% | 24.7% | 40.0% | 28.0% |
|  | 5b. Delivering organisation characteristics | 16.2% | 16.1% | 36.0% | 35.0% | 25.1% |
|  | 5c. Design: intervention treatment | 100.0% | 100.0% | 100.0% | 100.0% | 100.0% |
|  | 5d. Design: control treatment | 94.9% | 100.0% | 98.9% | 75.0% | 95.4% |
|  | 5e. Design: proscribed intervention components | 25.3% | 9.7% | 15.7% | 20.0% | 19.2% |
|  | 5f. Design: proscribed control components | 25.3% | 12.9% | 20.2% | 5.0% | 20.1% |
|  | 5g. Design: intervention materials | 67.7% | 71.0% | 92.1% | 50.0% | 75.7% |
|  | 5h. Design: control materials | 47.5% | 64.5% | 77.5% | 35.0% | 59.8% |
|  | 5i. Design: intervention format | 85.9% | 67.7% | 98.9% | 85.0% | 88.3% |
|  | 5j. Design: control format | 72.7% | 64.5% | 93.3% | 50.0% | 77.4% |
|  | 5k. Design: intervention duration | 89.9% | 96.8% | 92.1% | 100.0% | 92.5% |
|  | 5l. Design: control duration | 80.8% | 96.8% | 91.0% | 65.0% | 85.4% |
|  | 5m. Design: intervention frequency | 86.9% | 93.5% | 88.8% | 95.0% | 89.1% |
|  | 5n. Design: control frequency | 72.7% | 96.8% | 77.5% | 45.0% | 75.3% |
|  | 5o. Design: intervention intensity | 61.6% | 41.9% | 75.3% | 75.0% | 65.3% |
|  | 5p. Design: control intensity | 46.5% | 38.7% | 67.4% | 40.0% | 52.7% |
|  | 5q. Design: intervention staffing | 63.6% | 25.8% | 43.8% | 80.0% | 52.7% |
|  | 5r. Design: control staffing | 43.4% | 22.6% | 41.6% | 30.0% | 38.9% |
|  | 5s. Delivery: intervention treatment | 56.6% | 61.3% | 41.6% | 35.0% | 49.8% |
|  | 5t. Delivery: control treatment | 45.5% | 67.7% | 38.2% | 10.0% | 42.7% |
|  | 5u. Delivery: programme differentiation | 37.4% | 54.8% | 43.8% | 30.0% | 41.4% |
|  | 5v. Delivery: proscribed intervention components | 12.1% | 3.2% | 9.0% | 5.0% | 9.2% |
|  | 5w. Delivery: proscribed control components | 14.1% | 6.5% | 10.1% | 5.0% | 10.9% |
|  | 5x. Delivery: non-specific intervention components | 15.2% | 3.2% | 3.4% | 30.0% | 10.5% |
|  | 5y. Delivery: non-specific control components | 9.1% | 0.0% | 2.2% | 5.0% | 5.0% |
|  | 5z. Delivery: intervention materials | 55.6% | 67.7% | 91.0% | 40.0% | 69.0% |
|  | 5aa. Delivery: control materials | 40.4% | 64.5% | 76.4% | 25.0% | 55.6% |
|  | 5bb. Delivery: intervention format | 60.6% | 41.9% | 75.3% | 65.0% | 64.0% |
|  | 5cc. Delivery: control format | 48.5% | 35.5% | 73.0% | 30.0% | 54.4% |
|  | 5dd. Delivery: intervention duration | 71.7% | 87.1% | 88.8% | 70.0% | 79.9% |
|  | 5ee. Delivery: control duration | 62.6% | 80.6% | 88.8% | 40.0% | 72.8% |
|  | 5ff. Delivery: intervention frequency | 73.7% | 87.1% | 82.0% | 50.0% | 76.6% |
|  | 5gg. Delivery: control frequency | 56.6% | 80.6% | 78.7% | 25.0% | 65.3% |
|  | 5hh. Delivery: intervention intensity | 38.4% | 32.3% | 61.8% | 25.0% | 45.2% |
|  | 5ii. Delivery: control intensity | 26.3% | 22.6% | 58.4% | 10.0% | 36.4% |
|  | 5jj. Delivery: tailoring of intervention | 34.3% | 9.7% | 10.1% | 50.0% | 23.4% |
|  | 5kk. Delivery: tailoring of control | 17.2% | 3.2% | 5.6% | 15.0% | 10.9% |
|  | 5ll. Delivery: intervention staffing | 44.4% | 16.1% | 36.0% | 10.0% | 34.7% |
|  | 5mm. Delivery: control staffing | 26.3% | 12.9% | 34.8% | 5.0% | 25.9% |
|  | 5nn. Delivery: intervention provider training | 27.3% | 9.7% | 22.5% | 30.0% | 23.4% |
|  | 5oo. Delivery: control provider training | 19.2% | 3.2% | 18.0% | 10.0% | 15.9% |
|  | 5pp. Delivery: intervention supervision | 35.4% | 6.5% | 15.7% | 45.0% | 25.1% |
|  | 5qq. Delivery: control supervision | 22.2% | 0.0% | 12.4% | 15.0% | 15.1% |
|  | 5rr. Delivery: provider adherence measurement | 43.4% | 9.7% | 18.0% | 45.0% | 29.7% |
|  | 5ss. Delivery: participant compliance measurement | 34.3% | 12.9% | 19.1% | 30.0% | 25.5% |
|  | 5tt. Uptake: intervention treatment | 42.4% | 16.1% | 29.2% | 30.0% | 33.1% |
|  | 5uu. Uptake: control treatment | 34.3% | 19.4% | 27.0% | 15.0% | 28.0% |
|  | 5vv. Uptake: programme differentiation | 22.2% | 0.0% | 10.1% | 5.0% | 13.4% |
|  | 5ww. Uptake: contamination of intervention | 5.1% | 0.0% | 0.0% | 5.0% | 2.5% |
|  | 5xx. Uptake: contamination of control | 7.1% | 3.2% | 0.0% | 0.0% | 3.3% |
|  | 5yy. Uptake: proscribed intervention components | 3.0% | 0.0% | 0.0% | 0.0% | 1.3% |
|  | 5zz. Uptake: proscribed control components | 3.0% | 0.0% | 0.0% | 0.0% | 1.3% |
|  | 5aaa. Uptake: intervention materials | 36.4% | 41.9% | 67.4% | 15.0% | 46.9% |
|  | 5bbb. Uptake: control materials | 26.3% | 41.9% | 58.4% | 5.0% | 38.5% |
|  | 5ccc. Uptake: intervention frequency | 56.6% | 38.7% | 43.8% | 40.0% | 48.1% |
|  | 5ddd. Uptake: control frequency | 39.4% | 35.5% | 40.4% | 10.0% | 36.8% |
|  | 5eee. Uptake: intervention intensity | 24.2% | 19.4% | 30.3% | 30.0% | 26.4% |
|  | 5fff. Uptake: control intensity | 16.2% | 12.9% | 29.2% | 5.0% | 19.7% |
|  | 5ggg. Uptake: enactment | 27.3% | 3.2% | 14.6% | 25.0% | 19.2% |
|  | 6a. Baseline data collection | 80.8% | 61.3% | 55.1% | 60.0% | 66.9% |
|  | 6b. Baseline data measures | 80.8% | 38.7% | 65.2% | 60.0% | 67.8% |
|  | 6c. Primary outcome data collection | 49.5% | 25.8% | 32.6% | 15.0% | 37.2% |
|  | 6d. Primary outcome measures | 55.6% | 29.0% | 33.7% | 20.0% | 41.0% |
|  | 6e. Secondary outcome data collection | 83.8% | 93.5% | 83.1% | 90.0% | 85.4% |
|  | 6f. Secondary outcome measures | 94.9% | 87.1% | 86.5% | 95.0% | 90.8% |
|  | 6g. Period of follow-up | 98.0% | 100.0% | 94.4% | 100.0% | 97.1% |
|  | 6h. Methods to enhance quality of measurements | 66.7% | 51.6% | 62.9% | 55.0% | 62.3% |
|  | 6i. Changes to data collection protocol | 13.1% | 16.1% | 5.6% | 5.0% | 10.0% |
|  | 7a. Sample size calculation | 33.3% | 0.0% | 2.2% | 15.0% | 15.9% |
|  | 7b. Interim analyses and stopping rules | 2.0% | 0.0% | 0.0% | 5.0% | 1.3% |
|  | 8a. Random Sequence Generation | 33.3% | 16.1% | 7.9% | 30.0% | 21.3% |
|  | 8b. Randomisation restrictions | 44.4% | 16.1% | 34.8% | 55.0% | 38.1% |
|  | 8c. Allocation of providers | 20.2% | 3.2% | 28.1% | 5.0% | 19.7% |
|  | 9. Allocation concealment | 24.2% | 12.9% | 1.1% | 25.0% | 14.2% |
|  | 10a. Sequencer Generator | 22.2% | 9.7% | 1.1% | 25.0% | 13.0% |
|  | 10b. Who enrolled participants | 29.3% | 19.4% | 4.5% | 30.0% | 18.8% |
|  | 10c. Who assigned participants | 25.3% | 19.4% | 4.5% | 30.0% | 17.2% |
|  | 11a. Provider blinding | 6.1% | 0.0% | 5.6% | 20.0% | 6.3% |
|  | 11b. Participant blinding | 12.1% | 3.2% | 6.7% | 10.0% | 8.8% |
|  | 11c. Assessor blinding | 42.4% | 9.7% | 21.3% | 25.0% | 28.9% |
|  | 12a. Primary outcome analytic plan | 48.5% | 25.8% | 18.0% | 20.0% | 31.8% |
|  | 12b. Secondary outcome analytic plan | 68.7% | 64.5% | 44.9% | 85.0% | 60.7% |
|  | 12c. Sub-group and adjusted analyses | 56.6% | 32.3% | 24.7% | 60.0% | 41.8% |
| **Results** | 13a. Participant flow | 55.6% | 3.2% | 3.4% | 15.0% | 25.9% |
|  | 13b. Number approached | 56.6% | 16.1% | 9.0% | 40.0% | 32.2% |
|  | 13c. Number eligible | 57.6% | 16.1% | 7.9% | 45.0% | 32.6% |
|  | 13d. Number randomised | 73.7% | 45.2% | 75.3% | 75.0% | 70.7% |
|  | 13e. Treatment allocation | 48.5% | 9.7% | 16.9% | 40.0% | 31.0% |
|  | 13f. Attrition | 54.5% | 9.7% | 14.6% | 35.0% | 32.2% |
|  | 13g. Discontinued Intervention | 42.4% | 3.2% | 7.9% | 10.0% | 21.8% |
|  | 13h. Number in primary analysis | 54.5% | 12.9% | 28.1% | 40.0% | 38.1% |
|  | 14a. Period of recruitment | 36.4% | 16.1% | 2.2% | 45.0% | 21.8% |
|  | 14b. Recruitment process | 58.6% | 61.3% | 36.0% | 77.5% | 52.1% |
|  | 14.c Incentives | 46.5% | 41.9% | 33.7% | 50.0% | 41.4% |
|  | 14d. Reasons for stopping | 3.0% | 0.0% | 4.5% | 5.0% | 3.3% |
|  | 15. Baseline data | 64.6% | 16.1% | 29.2% | 65.0% | 45.2% |
|  | 16a. Number analysed | 68.7% | 22.6% | 64.0% | 70.0% | 61.1% |
|  | 16b. Intention-to-treat | 48.5% | 3.2% | 7.9% | 45.0% | 27.2% |
|  | 17a. Primary outcome results | 35.4% | 16.1% | 23.6% | 20.0% | 27.2% |
|  | 17b. Secondary outcome results | 45.5% | 22.6% | 49.4% | 55.0% | 44.8% |
|  | 18. Sub-group or adjusted analysis results | 61.6% | 38.7% | 46.1% | 55.0% | 52.3% |
|  | 19. Adverse events | 20.2% | 3.2% | 0.0% | 10.0% | 9.6% |
| **Discussion** | 20. Limitations | 90.9% | 67.7% | 67.4% | 95.0% | 79.5% |
|  | 21. Generalisability | 77.8% | 71.0% | 51.7% | 70.0% | 66.5% |
|  | 22a. Overall evidence | 90.9% | 87.1% | 80.9% | 70.0% | 84.9% |
|  | 22b. Results compared to hypotheses | 78.8% | 67.7% | 64.0% | 65.0% | 70.7% |
|  | 22c. Reference to systematic review | 74.7% | 51.6% | 46.1% | 45.0% | 58.6% |
|  | 22d. Reference to other studies | 55.6% | 16.1% | 13.5% | 65.0% | 35.6% |
| **Study Details** | 23. Trial registration | 10.1% | 0.0% | 0.0% | 5.0% | 4.6% |
|  | 24a. Protocol | 14.1% | 3.2% | 3.4% | 10.0% | 8.4% |
|  | 24b. Access to treatment manual | 47.5% | 32.3% | 32.6% | 45.0% | 39.7% |
|  | 25a. Conflicts of interest | 83.8% | 32.3% | 49.4% | 80.0% | 64.0% |
|  | 25b. Ethical considerations | 80.8% | 35.5% | 21.3% | 65.0% | 51.5% |
|  | 25c. Intervention development | 69.7% | 74.2% | 55.1% | 85.0% | 66.1% |

Number of RCTs in each discipline: RCTs per discipline: Clinical Psychology—99, Criminology—31, Education—89, Social Work—20

Number of standards in total score: 147
