# Supplementary material for: Reporting Quality of Social and Psychological Intervention Trials: A Systematic Review of Reporting Guidelines and Trial Publications
Source: PLoS One. 2013 May 29;8(5):e65442. doi: 10.1371/journal.pone.0065442 (PMC3666983; doi:10.1371/journal.pone.0065442)
Supplement: Text S1 — Electronic search strategy. (DOC) [file pone.0065442.s006.doc]

**Electronic Search Strategy adapted from [21]**

I. Ovid MEDLINE(R) –1948 to June Week 1 2011

1. exp Research Design/

2. exp Guideline/

3. exp Study characteristics/

4. exp Epidemiologic studies/

5. Feasibility Studies/

6. Intervention Studies/

7. Program Evaluation/

8. Evidence-Based Medicine/

9. Human Experimentation/

10. exp Research/

11. ((control$ or clinical or comparative$) adj2 (trial$ or stud$)).mp.

12. between group design$.mp.

13. random$.tw.

14. ((control$ or intervention or evaluation or comparative or effectiveness or evaluation or

feasibility) adj3 (trial or studies or study or program or design)).tw.

15. systematic review$.tw.

16. (time adj series).tw.

17. (pre test or pretest or posttest or post test).tw.

18. controlled before.tw.

19. or/1—18

20. (reporting and (trial$ or studies)).ti.

21. ((guideline$ or guide line$ or checklist$ or recommendation$ or standard$ or requirement$

or instruction$ or guidance$ or policies or policy) adj3 (reporting or publishing or good

practice or good practi#e$)).tw.

22. or/20—21

23. (good adj3 practi#e$ adj3 (reporting or publishing or publication)).tw.

24. (reporting adj2 (guideline or standard or standards or quality)).ti.

25. (19 and 22) or 23 or 24

26. limit 25 to english

II. Ovid EMBASE—1980-2011, Week 22

1. exp Research/

2. Epidemiology/

3. Randomized Controlled Trial/

4. exp Clinical Trial/

5. Meta Analysis/

6. Systematic Review/

7. Evidence Based Practice/

8. Practice Guideline/

9. or/1—8

10. (reporting and (trial$ or study or studies)).ti.

11. ((guideline$ or guide line$ or checklist$ or check list$ or recommendation$ or standard$ or

requirement$ or instruction$ or guidance$ or policies or policy) adj3 (reporting or

publishing or good practice or good practi#e$)).tw.

12. (or/10—11) and 9

13. (reporting adj2 (guideline or standard or standards)).ti.

14. (good adj3 practi#e$ adj3 (reporting or publishing or publication)).tw.

15. or/12—14

16. limit 15 to english

C. Ovid PsycINFO—1806 to June, Week 1, 2011

1. Treatment Effectiveness Evaluation/
2. exp Clinical Trial/
3. Meta Analysis/
4. exp Experimentation/
5. Evidence Based Practice/
6. exp Experimental Design/
7. Methodology/
8. or/1—7
9. (reporting and (trial$ or studies or study)).ti.
10. ((guideline$ or guide line$ or checklist$ or check list$ or recommendation$ or standard$ or requirement$ or instruction$ or guidance$ or policies or policy) adj3 (reporting or publishing or good practice or good practi#e$)).tw.
11. or/9—10
12. (reporting adj2 (guideline or standard or standards)).ti.
13. (good adj3 practi#e$ adj3 (reporting or publishing or publication)).tw.
14. (11 and 8) or 12 or 13
15. limit 14 to english

D. Cochrane Methodology Register—Cochrane Library 2011

Record title:

(reporting near/2 (guideline or standard or standards))

OR

Search all text:

((guideline* or checklist* or recommendation* or standard* or requirement* or instruction* or guidance* or policies or policy) near/3 (reporting or publishing or good practi#e*))

OR

Keywords:

(checklists and guidelines)

E. Scopus Social Sciences—1966 to June, Week 1 2011

TITLE-ABS-KEY((reporting W/2 guideline) OR (reporting W/2 standard*) OR (reporting near/2 quality))

F. ISI Web of Knowledge Social Science Citation Index—1945 to June 7 2011

TS=((reporting near/2 guideline) OR (reporting near/2 standard*) OR (reporting near/2 quality))
